# Genetic Prediction of Antidepressant Drug Response and Nonresponse in Korean Patients

**DOI:** 10.1371/journal.pone.0107098

**Published:** 2014-09-16

**Authors:** Shinn-Won Lim, Hong-Hee Won, Hyeran Kim, Woojae Myung, Seonwoo Kim, Ka-Kyung Kim, Bernard J. Carroll, Jong-Won Kim, Doh Kwan Kim

**Affiliations:** 1 Center for Clinical Research, Samsung Biomedical Research Institute, Seoul, Korea; 2 Department of Psychiatry, Samsung Medical Center, Sungkyunkwan University School of Medicine, Seoul, Korea; 3 Biostatistics Unit, Samsung Biomedical Research Institute, Seoul, Korea; 4 Department of Laboratory Medicine and Genetics, Samsung Medical Center, Sungkyunkwan University School of Medicine, Seoul, Korea; 5 Pacific Behavioral Research Foundation, Carmel, California, United States of America; Radboud University, Netherlands

## Abstract

Genetic polymorphism contributes to variation in response to drug treatment of depression. We conducted three independent 6-week treatment studies in outpatients with major depressive disorder (MDD) to develop a pharmacogenomic model predicting response and nonresponse. We screened candidate genomic markers for association with response to selective serotonin reuptake inhibitors (SSRIs). No patients had received any antidepressant drug treatment in the current episode of depression. Outcome evaluation was blinded to drug and genotype data. The prediction model derived from a development sample of 239 completer cases treated with SSRIs comprised haplotypes and polymorphisms related to serotonin synthesis, serotonin transport, glutamate receptors, and GABA synthesis. The model was evaluated prospectively for prediction of outcome in a validation sample of 176 new SSRI-treated completer cases. The model gave a prediction in 60% of these cases. Predictive values were 85% for predicted responders and 86% for predicted nonresponders, compared to prior probabilities of 66% for observed response and 34% for observed nonresponse in those cases (both *P*<0.001). Convergent cross-validation was obtained through failure of the model to predict outcomes in a third independent sample of 189 completer cases who received non-SSRI antidepressants. We suggest proof of principle for genetic guidance to use or avoid SSRIs in a majority of Korean depressed patients.

## Introduction

Response rates in drug treatment of major depression are variable and often less than 50% in “real world” studies [1], and there are no biomarkers to direct choice of antidepressant drug class. Genetic markers hold promise for improving this record [2]–[4].

Many studies have focused on a few genes related to the primary actions of the drugs. Genetic polymorphism in the serotonin transporter (5-hydroxytryptamine transporter, 5-HTT), has been linked to antidepressant response to selective serotonin reuptake inhibitors (SSRIs) [5]–[7], although not in all studies [8]. Among the factors affecting functional response to antidepressant drugs are multiple secondary neurobiological mechanisms, environmental factors, ethnicity, and drug class. Based on our earlier reports [5], [9], we adopted an expanded survey of candidate genes using single nucleotide polymorphism (SNP) microarray methods. Candidate genes were selected for the primary targets and secondary mechanisms affected by antidepressant drugs. We used a candidate gene strategy rather than an exploratory genome wide association study (GWAS) which requires much larger sample sizes [10].

**Figure 1:**
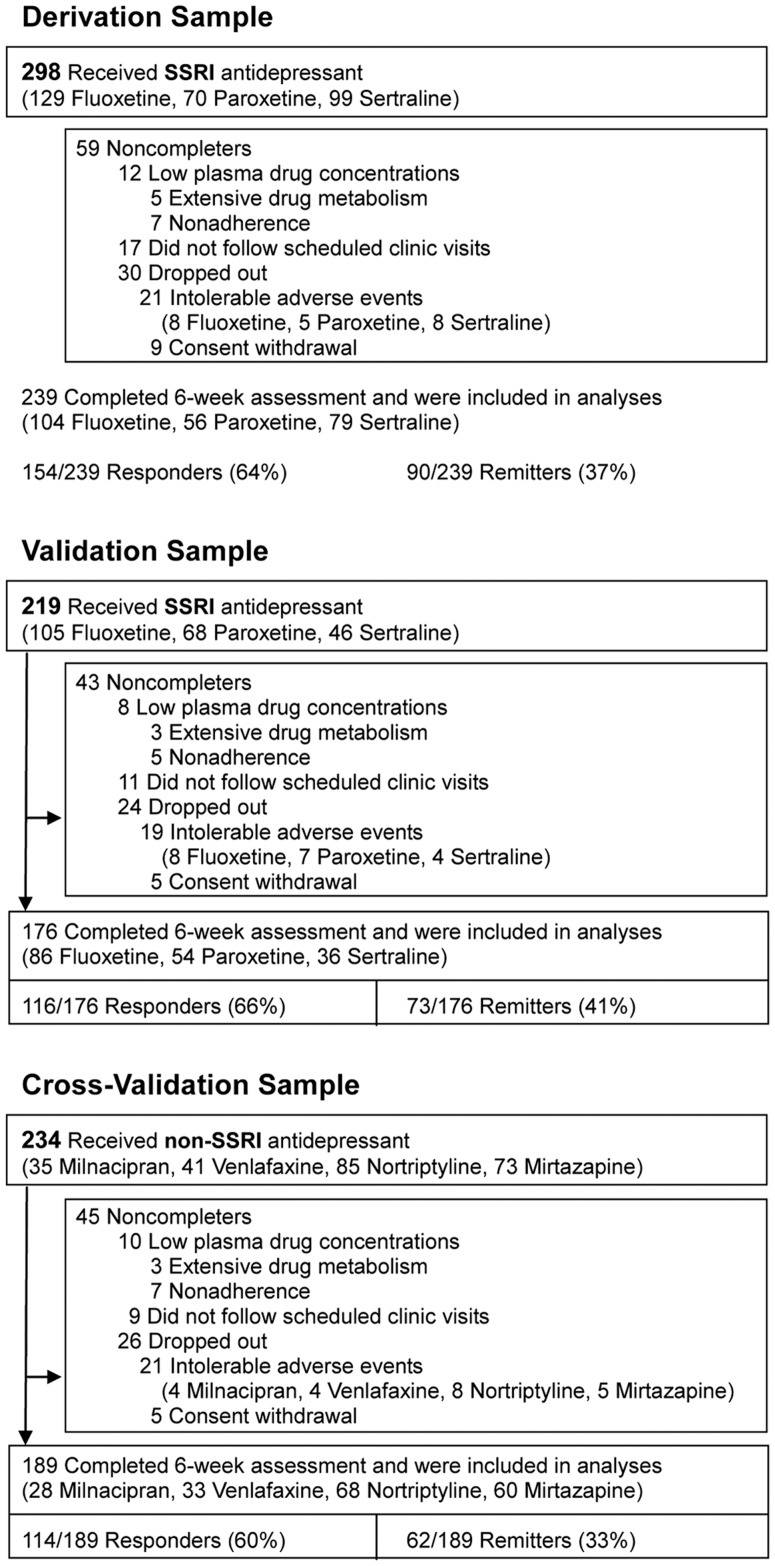
Enrollment, attrition, drug treatments, and outcomes of patients in all samples.

Here we report on a 3-stage study (Figure 1) of multiple candidate genes for predicting response and nonresponse to SSRIs in depressed patients. After identifying a predictive model for SSRI response in the derivation sample, we subjected the model to validation testing in the second, independent, sample of patients, who also received SSRI treatment. The cross-validation sample, also independent, was treated with non-SSRI drugs. This third sample served 3 purposes – as a partial solution to the absence of a placebo-treated group; to evaluate whether the predictive SSRI model generalized to another class of antidepressant drug; and for exploration of gene markers of response to non-SSRI agents. We hypothesized that the predictive model for SSRI response would predict response to SSRI treatment in the validation sample, whilst it would not predict response to non-SSRI drugs in the cross-validation sample. These predictions are consistent with other reports of drug class differences [9], [11].

## Methods

### Participants

We studied 3 independent cohorts totaling 751 Korean adult outpatients with major depression. These samples were completely separate from our previous reports [5], [9]. The first (derivation) sample (N = 298) received SSRI drugs. The second (validation) sample (N = 219) also received SSRI drugs. The third (cross-validation) sample (N = 234) received non-SSRI drugs. No patients had received any antidepressant drug treatment during the current episode of depression. The study was conducted in a naturalistic clinical setting rather than in a placebo-controlled clinical trial [12], [13]. The protocol was approved by the ethics review board of Samsung Medical Center, Seoul, Korea. Signed informed consent was obtained from all participants. The study is registered (NCT00817375) in ClinicalTrials.gov.

The assessment and quality control procedures have been fully described previously [5], [9]. A total 782 participants were recruited from patients seeking care for depression at a university hospital from October 1997 through July 2007. Thirty-one cases were excluded: 6 patients did not have a significant other to obtain collateral diagnostic information, 4 patients had significant medical conditions, 7 patients had a concomitant Axis I psychiatric disorder, and 16 patients did not have a minimum 17-item HAM-D score of 15. Two cases met 2 of these exclusion criteria. Thus, a total of 751 Korean outpatients with MDD were enrolled. All were clinically referred and all were of unrelated Korean ancestry. Figure 1 displays retention and attrition data for the 3 independent clinical samples. As this is a discovery project, outcome analyses included only subjects who completed 6 weeks of treatment with adequate blood levels (Figure 1). Extensive drug metabolizers were distinguished from nonadherent cases by clinical review after a low blood level was detected. Overall, 604 patients (80.4%) completed the protocol.

Enrolled patients met the *Diagnostic and Statistical Manual of Mental Disorders, Fourth Edition* (DSM-IV) criteria for MDD without psychotic features. The diagnosis was based on an initial clinical interview, followed by a structured research interview, the Samsung Psychiatric Evaluation Schedule (SPES). The affective disorder section of the SPES uses the Korean version of the *Structured Clinical Interview for the Diagnostic and Statistical Manual of Mental Disorders, Fourth Edition*. The SPES provides additional information including cognitive screening, comorbid psychiatric diagnoses, psychosocial variables (age, sex, age of onset, duration of current episode, episode number), family history and initial Hamilton Depression Rating Scale (HAM-D) [14] severity score. These diagnostic interviews involved the patient and at least one family member. The final diagnosis was made after review of ongoing clinical observations, medical records, past histories, and the DSM-IV criteria, by a board-certified psychiatrist. Inclusion criteria were 18 years of age or older, the existence of a current nonpsychotic unipolar major depressive episode as verified by DSM-IV criteria, a minimum 17-item HAM-D score of 15, and ability to provide informed consent. To be included in these discovery analyses, patients also were required to adhere to prescribed medication and to have an adequate plasma antidepressant drug level measured at 6 weeks. Exclusion criteria were pregnancy, significant medical conditions, abnormal baseline laboratory values, unstable psychiatric features (e.g., suicide attempt), histories of alcohol or drug dependence, seizure disorder, neurological illness including significant cognitive impairment, or concomitant Axis I psychiatric disorder. Patients with MDD who met DSM-IV criteria for the specifier ‘*Severe With Psychotic Features*’ were excluded because they would normally receive concurrent antipsychotic medication. As stated above, no patients had received antidepressant drug treatment in the current episode before enrolment in this study. In addition, no patient had received non-antidepressant psychotropic medication within 2 weeks of the study. We also verified that no patients had received fluoxetine, which has a long half-life, for any reason within the preceding 4 weeks.

### Procedures

Patients received monotherapy for 6 weeks with one of three commonly used SSRI drugs or one of four non-SSRI antidepressants, by clinician's choice (Figure 1). In this naturalistic study, choice of drug was driven by the preference of the physician, with consideration of anticipated side effects in at-risk individuals [15]. Dose titration was completed within two weeks. Trough plasma samples were drawn at the end of week 6 for plasma drug concentrations. Lorazepam 0.5–1 mg was allowed at bedtime for insomnia. Patients were seen by a psychiatrist, who monitored their adverse events by the Udvalg for Kliniske Undersogelser (UKU) scale [16] at weeks 0, 1, 2, 4, and 6. The 17-item Hamilton scale for depression (HAM-D) [14] was administered by a single trained rater every two weeks. The rater and genotyper were blinded to the hypotheses and to drug assignment. HAM-D and genotype data were not disclosed to the psychiatrist, and the rater was blinded to the genotype data. To maintain the blindness, a trained research coordinator managed the data and schedules. At six weeks, response was defined according to standard conventions [4] as ≥50% decrease in the HAM-D score, and remission as a HAM-D score ≤7.

The protocol completion rates were 80% (derivation sample), 80% (validation sample), and 81% (cross-validation sample) (Figure 1). For comparison, protocol completion rates in controlled clinical trials of antidepressant drugs typically are 70–75% [17]. As shown in Figure 1, dropouts occurred for the usual clinical and administrative reasons, and we excluded cases with evidence of nonadherence or extensive drug metabolism inferred from the finding of low plasma drug concentrations at week 6. The clinical characteristics of non-completers did not differ significantly from completers in any cohort (data not shown). The data reported in the Results apply to the 604 completer cases: 239 in the derivation cohort; 176 in the validation SSRI cohort; and 189 in the cross-validation non-SSRI cohort (Figure 1).

### Candidate genes and selection of SNP markers

We focused on candidate genes of neurotransmitter metabolic enzymes, transporters and receptors (Table S1). We selected 79 candidate genes, based on their likely importance for immediate or delayed mechanisms of antidepressant action. We combined knowledge-based and function-based tagging selection approaches (Figure S1). We selected 155 SNPs through a literature survey on the significant SNPs related to antidepressant response, and 1657 SNPs by tagging based on potential functional importance (Table S2).

After screening for availability of Golden Gate Bead Array analysis (Illumina, Inc. San Diego, CA), 1502 SNPs were genotyped. 67 SNPs with a call rate of less than 95% and 35 SNPs with a minor allele frequency less than 5% were excluded. Finally, 1400 SNPs were prepared.

To enable comparison with SNP data using different SNP genotyping platforms, we imputed genotypes for untyped SNPs using the IMPUTE software [18].

### Statistical analysis

We performed tests of five genetic modes (dominant, recessive, genotype, allele, and additive) for each SNP with the use of Fisher's exact test and the Cochran-Armitage test [19]. The mode most strongly associated with response was considered the best-fitting genetic mode for each SNP. These significance levels were calculated and corrected with the false discovery rate (FDR) control [20].

Haplotype blocks were defined in the derivation sample by confidence intervals using Haploview [21], [22]. Associations between haplotype blocks and response were tested using Fisher's exact test with the FDR control. Multivariable analyses for SNPs and for haplotype blocks found to be significant in univariable analyses were performed using multiple logistic regression and the Generalized Estimating Equations method [23], respectively.

Prediction models for response and nonresponse were constructed using multiple logistic regression. We constructed two types of prediction model. First, only polymorphic markers were considered (polymorphism model, section 4 of Text S2). Second, in addition to SNPs and VNTR markers, haplotypes were included and considered in the model (HAP-SNP model). Before constructing a combined haplotype-SNP (HAP-SNP) model, haplotypes were re-defined as a pair of two haplotypes (for example, *TPH2* H3-A is defined as a pair of two haplotypes (GCATGG and GCATGG) because the haplotypes are clustered data. We used the operational criteria of probability>0.8 for predicting response (better than the optimal response rate expected with combined drug and cognitive behavioral therapy in common psychiatric disorders such as depression and anxiety) [24], [25], and response probability <0.3 for predicting nonresponse (lower than conservative estimates of the expected response rate with placebo in controlled clinical trials for depression [26]). This approach stratified each sample as predicted responders, predicted nonresponders, and indeterminate cases (no prediction). Excluding the indeterminate cases, we calculated overall accuracy, positive predictive value (PPV), negative predictive value (NPV), sensitivity and specificity, and areas under the receiver operating curve (AUC). The significance of the change from prior probabilities in the absence of genotyping to posterior probabilities from the prediction model was tested by the Chi square Goodness of Fit method. The PPVs and NPVs between the derivation set and the validation set were compared by Fisher's exact test.

The study was powered for the outcomes of observed response and nonresponse (see Text S1, section 4). All p values were reported as two-sided, and *P* values <0.05 were considered statistically significant. Analyses were performed with the use of the SAS software, version 9.13.

Detailed methods of function-based tagging selection of SNP markers, genotyping, power analysis and quantification of plasma drug levels are described in Text S1 (Supplementary Method).

## Results

### Clinical characteristics

Demographic variables, response and remission rates, severity ratings, and salient clinical variables of the three samples are shown in Table 1. HAM-D scores indicated moderate to severe depression. Observed response rates exceeded 60% in all groups. Responders and nonresponders did not differ at baseline on any variable, except for duration of episode in the derivation sample. Choice of SSRI drug did not influence outcomes overall (response rate to fluoxetine, paroxetine and sertraline: 65.4%, 64.3% and 63.3%, see Table S3) or in relation to any genotype (Table S3). Plasma drug levels in responders and nonresponders were not significantly different (Table S4).

**Table 1:** Characteristics of patients in derivation sample, validation sample, and cross-validation sample.

	Derivation Sample			Validation Sample				Cross-Validation Sample			
Characteristics	SSRI Treated Group			SSRI Treated Group			*P* +	Non-SSRI Treated Group			*P* ++
	Responder	Nonresponder	*P*	Responder	Nonresponder	*P*		Responder	Nonresponder	*P*	
Response Rate (%)^a^	154/239 (64.4%)			116/176 (65.9%)			1.00	114/189 (60.3%)			0.84
Remission Rate (%)^a^	90/239 (37.7%)			73/176 (41.5%)			0.96	62/189 (32.8%)			0.62
Gender, Female (%)^a^	180/239 (75.3%)			136/176 (77.3%)			1.00	137/189 (72.5%)			1.00
	119/154 (66.1%)	61/85 (71.8%)	0.35	91/116 (78.5%)	45/60 (75.0%)	0.70		83/114 (72.8%)	54/75 (72.0%)	1.00	
Age, Mean±SD (Range), Year^b^	59.8±13.7 (19–86)			62.3±12.7 (24–87)			0.11	58.7±13.3 (22–82)			0.82
	59.1±14.0 (19–86)	61.1±13.2 (24–85)	0.35	62.0±12.3 (27–87)	62.7±13.4 (24–83)	0.47		58.7±13.0 (22–82)	59.4±13.9 (25–82)	0.21	
Family History of Depression (%)^a^	46/239 (19.3%)			34/176 (19.3%)			1.00	39/189 (20.6%)			1.00
	33/154 (21.4%)	13/85 (15.3%)	0.30	23/116 (19.8%)	11/60 (18.3%)	1.00		23/114 (20.2%)	16/75 (21.3%)	0.86	
Duration of Index Episode, Mean±SD (Range), Month^b^	9.6±14.1 (1–92)			8.8±13.2 (1–92)			1.00	10.8±15.9 (1–120)			0.82
	9.1±15.3 (1–92)	10.5±11.4 (1–71)	0.04	8.2±12.1 (1–63)	10.0±15.2 (1–92)	0.36		11.1±18.7 (1–120)	10.4±10.5 (1–37)	0.13	
No. of Episodes, Mean±SD (Range)^b^	2.2±1.8 (1–16)			2.3±2.0 (1–16)			1.00	2.2±2.1 (1–19)			1.00
	2.2±2.0 (1–16)	2.1±1.2 (1–6)	0.31	2.3±2.2 (1–16)	2.2±1.8 (1–12)	0.86		2.1±1.8 (1–11)	2.4±2.4 (1–19)	0.20	
Age at First Episode, Mean±SD (Range), Year^b^	53.0±15.1 (16–82)			55.6±14.5 (15–82)			0.18	51.2±15.0 (15–77)			0.56
	52.4±15.7 (16–81)	54.3±13.9 (19–82)	0.47	55.4±14.4 (20–81)	56.0±15.0 (15–82)	0.66		51.5±14.4 (15–77)	50.9±15.9 (15–75)	0.90	
HAM-D Baseline, Mean±SD (Range)^b^	20.9±4.8 (15–40)			19.2±3.4 (15–32)				21.5±5.1 (15–40)			
	20.8±5.3 (15–40)	21.0±4.0 (15–31)	0.45^c^	19.1±3.2 (15–29)	19.4±3.7 (15–32)	1.00		21.4±5.1 (15–40)	21.6±5.2 (15–36)	1.00^c^	
HAM-D After, Mean±SD (Range)^b^	10.2±5.6 (0–26)			9.5±5.3 (0–30)				11.7±6.5 (1–35)			
	7.0±3.1 (0–15)	16.0±4.3 (9–26)	<0.01^c^	6.5±2.8 (0–14)	15.3±4.0 (9–30)	<0.01		7.4±3.1 (1–17)	18.1±4.6 (10–35)	<0.01^c^	

Abbreviations: SSRI, selective serotonin reuptake inhibitor; HAM-D, Hamilton depression rating score.

a Fisher's exact test.

b Mann-Whitney test.

c Corrected by Bonferroni's correction for multiple testing.

+ Comparison between derivation sample and validation sample.

++ Comparison between derivation sample and cross-validation sample.

### Significant polymorphic markers for SSRI response

In the derivation sample, ten of 1400 candidate SNPs showed significant associations (*P*<0.05) with response after FDR correction (Table 2). These resided in four genes: four in *TPH2*, two in *GRIK2*, two in *GAD1*, and two in *SLC6A4*. The *TPH2* gene was most strongly associated with SSRI response. The rs4760815 in intron 6 of *TPH2* showed the strongest association (*P* = 1.26×10^−5^), and rs11179027, rs17110532 and rs17110747 in *TPH2* were also significantly associated (*P* = 1.57×10^−5^, 8.86×10^−5^ and 1.94×10^−4^).

**Table 2:** The SNPs most strongly associated with SSRI response (*P* <0.05 after FDR correction) and the strongly associated polymorphisms in *SLC6A4* from our previous study [9].

Gene	Chromosome	Position^a^	SNP	Responsive Allele	RAF in Responders	RAF in Nonresponders	MAF	*P* Value^b^	*P* Value by Bonferroni's Correction	*P* Value by Controlling FDR	Genetic Mode	Heterozygote Odds Ratio (95% CI)	Homozygote Odds Ratio (95% CI)
*TPH2*	12	70658496	rs4760815	T	0.60	0.41	0.50	1.26×10^−5^	0.02	0.02	Dominant	3.77 (3.55–4.00)	4.39 (2.08–9.29)
*TPH2*	12	70663579	rs11179027	C	0.55	0.34	0.44	1.57×10^−5^	0.02	0.02	Allele	2.69 (1.45–4.99)	4.77 (2.17–10.49)
*GRIK2*	6	102158042	rs543196	C	0.65	0.46	0.44	4.84×10^−5^	0.07	0.02	Additive	1.69 (0.83–3.45)	5.02 (2.18–11.53)
*GAD1*	2	171390986	rs3828275	G	0.72	0.64	0.31	6.89×10^−5^	0.10	0.02	Genotype	0.31 (0.17–0.55)	1.24 (0.43–3.62)
*TPH2*	12	70650935	rs17110532	C	0.42	0.24	0.33	8.86×10^−5^	0.12	0.02	Allele	2.02 (1.14–3.59)	5.36 (1.93–14.87)
*SLC6A4*	17	25575791	rs2066713	C	0.96	0.86	0.07	1.26×10^−4^	0.18	0.03	Recessive	0.48 (0.03–8.42)	2.27 (0.14–36.87)
*GRIK2*	6	102157181	rs572487	G	0.59	0.41	0.48	1.36×10^−4^	0.19	0.03	Additive	1.65 (1.54–1.77)	4.76 (2.09–10.86)
*TPH2*	12	70712221	rs17110747	A	0.31	0.16	0.25	1.94×10^−4^	0.27	0.03	Allele	2.53 (1.37–4.69)	3.88 (1.25–11.99)
*GAD1*	2	171379072	rs12185692	C	0.71	0.65	0.31	2.33×10^−4^	0.33	0.04	Genotype	0.35 (0.20–0.62)	1.57 (0.49–5.03)
*SLC6A4*	17	25571040	rs2020942	G	0.95	0.85	0.09	2.96×10^−4^	0.42	0.04	Additive	1.27 (1.21–1.34)	4.56 (0.41–51.22)

Gene	Chromosome		VNTR		*P* Value^b^	*P* Value by Bonferroni's Correction	*P* Value by Controlling FDR	Genetic Mode	Odds Ratio (95% CI)
*SLC6A4*	17		*5-HTT* VNTR in promoter (*5-HTTLPR*)		6.00×10^−3^	NA	NA	*ss* vs. *sl*+*ll*	2.18 (1.27–3.75)
*SLC6A4*	17		*5-HTT* VNTR in intron 2 (*STin2*)		2.00×10^−4^	NA	NA	*ll* vs. *sl*+*ss*	3.86 (1.90–7.84)

Abbreviations: SNP, single-nucleotide polymorphism; SSRI, selective serotonin reuptake inhibitor; RAF, responsive allele frequency; FDR, false discovery rate; VNTR, variable number of tandem repeat; MAF, minor allele frequency; NA, not applicable.

a Genomic position (NCBI Build 36).

b Fisher's exact test.

The second strongest associations with response to SSRIs were found in rs543196 and rs572487 in intron 2 of *GRIK2* (*P* = 4.84×10^−5^ and 1.36×10^−4^). Another strong association was found in *GAD1*, where rs3828275 in intron 3 and rs12185692 located ∼2.5 kb upstream of this gene showed strong association (*P* = 6.89×10^−5^ and 2.33×10^−4^).

Two SNPs, rs2066713 and rs2020942, in the serotonin transporter gene (*SLC6A4*) also showed strong association with SSRI response (*P* = 1.26×10^−4^ and 2.96×10^−4^). Previously, we reported that 44 bp insertion/deletion polymorphisms in the promoter region (*5-HTTLPR*) and variable number of tandem repeat (VNTR) *s/l* polymorphisms in intron 2 (*STin2*) of *SLC6A4* were associated with response to SSRIs [5], [9]. We also genotyped these two VNTRs, and they once again showed significant associations with response to SSRIs (*P*<0.01) (Table 2).

### Haplotype analysis for SSRI response

**Figure 2:**
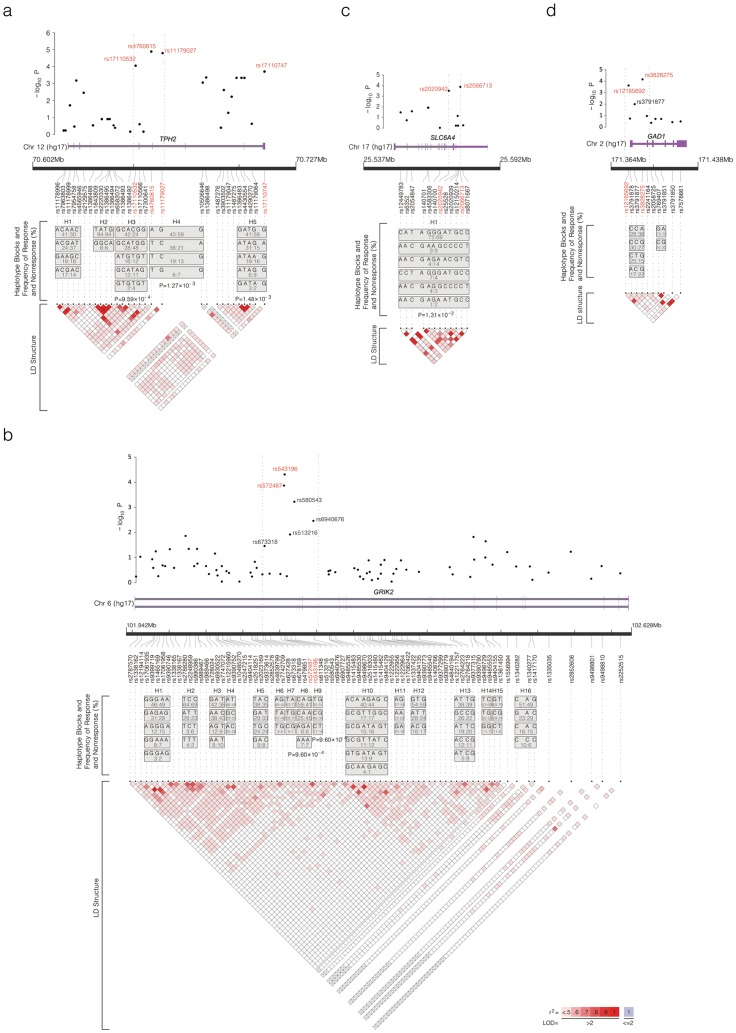
Linkage disequilibrium (LD) and haplotype structure of (a) *TPH2*, (b) *GRIK2*, (c) *SLC6A4*, and (d) *GAD1*. The LD structure in the lower panel is based on the measure of *r^2^*. Dark red indicates strong LD between two markers with high *r^2^* and a logarithm of odds (LOD) score of greater than 2.0. Haplotype frequencies of responders and nonresponders are also shown in each box in order (responders: nonresponders). The figure was prepared with LocusView2.0 (http://www.broad.mit.edu/mpg/locusview). Significant SNPs inscribed in red are plotted with their association analysis p values (as –log_10_ values) in the upper panel. Haplotype blocks and estimated haplotypes for each block are presented in the middle panel. (a) Among 30 SNPs screened in *TPH2*, four SNPs (colored red), rs17110532, rs4760815, rs11179027 and rs17110747, were significantly associated (see Table 2). The third (H3), fourth (H4) and fifth haplotype (H5) blocks were significantly associated with drug response (*P*<0.05 after FDR correction). (b) Among 78 SNPs in *GRIK2*, two SNPs (colored red), rs543196 and rs572487, were significantly associated. Four SNPs, rs580543, rs6940676, rs513216, and rs673318, adjacent to the peak SNPs also showed high associations. The eighth (H8) and ninth (H9) blocks were significantly associated with response (*P*<0.05 after FDR correction). (c) Among 12 SNPs in *SLC6A4*, two SNPs (colored red), rs2066713 and rs2020942, were significantly associated with response. The first haplotype (H1) block was significantly associated with response (*P*<0.05 after FDR correction). (d) Among ten SNPs in *GAD1*, two SNPs (colored red), rs3828275 and rs12185691, were significantly associated (see Table 2). No haplotype blocks were significantly associated with response.

We further analyzed the four major genes (*TPH2*, *GRIK2*, *GAD1* and *SLC6A4*) which have multiple significant SNPs by examining linkage disequilibrium (LD) structures and haplotypes. Six haplotype blocks in those genes except *GAD1* were significantly associated with SSRI response (FDR corrected *P*<0.05). Among five haplotype blocks observed in *TPH2*, the third (H3), fourth (H4) and fifth (H5) blocks were significantly associated with response (*P*<0.01) (Figure 2A). When we examined haplotypes and LD structure separately for the responders and nonresponders to SSRI drugs, LD was stronger and haplotype blocks were longer in the responders than the nonresponders (Figure S2).

Among 16 haplotype blocks constructed from 78 SNPs of *GRIK2*, the eighth (*P* = 9.6×10^−4^) and ninth (*P* = 9.6×10^−4^) haplotype blocks were significantly associated with SSRI response (Figure 2B). Only one haplotype block from 12 SNPs of *SLC6A4* was significantly associated (*P* = 1.3×10^−2^) (Figure 2C). However, two haplotype blocks from ten SNPs of *GAD1* were not significantly associated with response to SSRI drugs (Figure 2D).

### Prediction model for SSRI response

Using the stated operational criteria for predicting observed response and nonresponse to SSRIs (see Method; statistical analysis), the two prediction models demonstrated similar predictive performance. The HAP-SNP model made predictions for 54% of cases (129/239), compared with 46% (110/239) of patients using the polymorphism model. For this reason, we report on the HAP-SNP model as the optimal prediction model for response to SSRI treatment in this study. Genotypic combinations of the HAP-SNP model are presented in Table 3.

**Table 3:** Genotypic combinations of haplotype-SNP (HAP-SNP) prediction model.

	TPH2 (H3)*	SLC6A4 (H1)†	rs543196	rs3828275	5-HTTLPR	
Predicted responder	H3-B	H1-A	CC	AA	ss	>80% (n = 90)
	H3-B	H1-A	CC	GG	ss	
	H3-B	H1-A	CC	AA	sl+ll	
	H3-B	H1-A	TC	AA	ss	
	H3-B	H1-A	CC	GG	sl+ll	
	H3-B	H1-A	TC	GG	ss	
	H3-B	H1-A	CC	AG	ss	
	H3-B	H1-A	TC	AA	sl+ll	
Predicted Nonresponder	H3-B	H1-B	TT	GG	ss	<30% (n = 39)
	H3-B	H1-B	TC	AG	ss	
	H3-A	H1-A	TT	AA	ss	
	H3-A	H1-B	CC	AA	ss	
	H3-A	H1-A	TC	GG	sl+ll	
	H3-A	H1-A	CC	AG	sl+ll	
	H3-B	H1-B	TT	AA	sl+ll	
	H3-A	H1-A	TT	GG	ss	
	H3-A	H1-A	TC	AG	ss	
	H3-A	H1-B	CC	GG	ss	
	H3-B	H1-B	TT	GG	sl+ll	
	H3-B	H1-B	TC	AG	sl+ll	
	H3-A	H1-A	TT	AA	sl+ll	
	H3-B	H1-B	TT	AG	ss	
	H3-A	H1-B	CC	AA	sl+ll	
	H3-A	H1-B	TC	AA	ss	
	H3-A	H1-A	TT	GG	sl+ll	
	H3-A	H1-A	TC	AG	sl+ll	
	H3-A	H1-B	CC	GG	sl+ll	
	H3-A	H1-A	TT	AG	ss	
	H3-A	H1-B	TC	GG	ss	
	H3-A	H1-B	CC	AG	ss	
	H3-B	H1-B	TT	AG	sl+ll	
	H3-A	H1-B	TC	AA	sl+ll	
	H3-A	H1-B	TT	AA	ss	
	H3-A	H1-A	TT	AG	sl+ll	
	H3-A	H1-B	TC	AA	sl+ll	
	H3-A	H1-B	CC	AG	sl+ll	
	H3-A	H1-B	TT	GG	ss	
	H3-A	H1-B	TC	AG	ss	
	H3-A	H1-B	TT	AA	sl+ll	
	H3-A	H1-B	TT	GG	sl+ll	
	H3-A	H1-B	TC	AG	sl+ll	
	H3-A	H1-B	TT	AG	ss	
	H3-A	H1-B	TT	AG	sl+ll	

Abbreviations: SNP, single-nucleotide polymorphism; *5-HTTLPR,* serotonin-transporter-linked polymorphic region.

*H3-A is defined as a pair of two haplotypes (GCATGG and GCATGG), and H3-B as the other cases.

† H1-A is defined as any pairs constituting of the CATAGGGATGCC, CATAGGGACGCC, CATAGGAACGTC, CCTAGGGATGCC, AATAGGGATGCC, AACGAGGCCCCT, AACGAGAATGCC and AACGAAGCCCCT haplotypes, and H1-B as any pairs including at least one haplotype among the AACGAGAACGTC, CATAGGGCCCCC and CATGAGGATGCC haplotypes.

Moreover, we examined the effect of duration of episode, which differed between responders and nonresponders (Table 1), in the modeling but found that this clinical feature did not contribute significantly.

**Figure 3:**
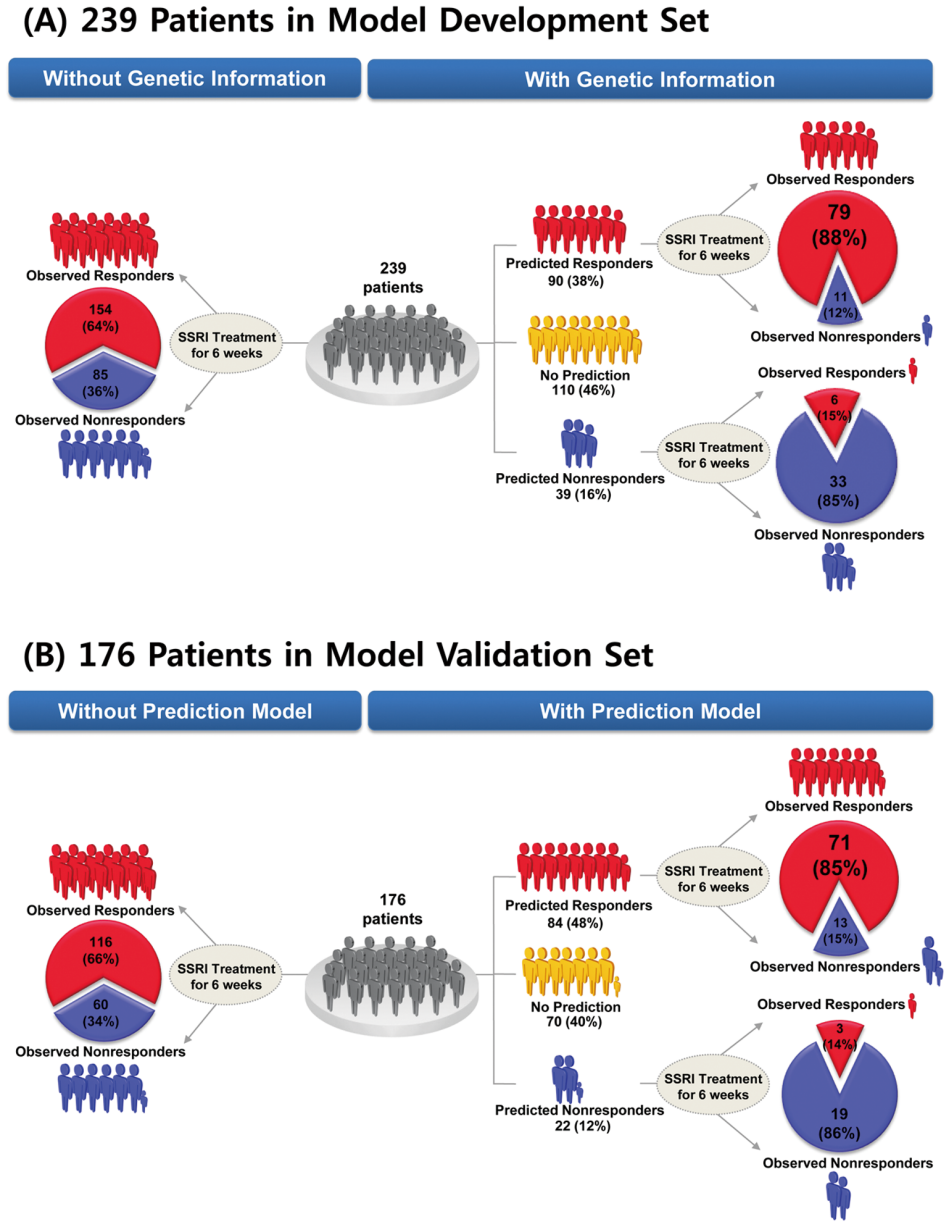
Clinical performance of selective serotonin reuptake inhibitor (SSRI) response prediction model using genetic information. Results of genetic prediction of response or nonresponse to SSRIs (a) in initial derivation sample (n = 239) and (b) validation sample (n = 176) of completer patients with major depression.

The HAP-SNP model contained polymorphic markers and haplotype blocks: *TPH2* (H3) (*P*<0.001), *SLC6A4* (H1) (*P*<0.001), rs543196 of *GRIK2* (*P*<0.001), rs3828275 of *GAD1* (*P* = 0.01), and *5-HTTLPR* of *SLC6A4* (*P* = 0.04), and showed an AUC of 0.82, which is considered an overall good performance [27]. The model predicted outcome for 54% of completer cases (129/239) in the derivation sample, with 90 predicted responders (>80% predicted probability of response) and 39 predicted nonresponders (<30% predicted probability of response) (Figure 3A). The observed outcomes in these 129 cases were 85 responders and 44 nonresponders (observed response rate 66%). For these 129 cases, 79 of 85 observed responders were correctly predicted (sensitivity 93%; 95% confidence interval [CI] 88%–98%), as were 33 of 44 observed nonresponders (specificity 75%; [62%–88%]). The positive predictive value (PPV) was 88% (79/90; [81%–95%]) and the negative predictive value (NPV) was 85% (33/39; [74%–96%]). The overall accuracy of prediction was 112 of 129 predicted cases (87%; [81%–93%]). The prior probabilities of observed response (66%) and nonresponse (34%) in the absence of genotyping increased to posterior probabilities of 88% and 85%, respectively (*P*<0.001 in each case). For the remaining 110 cases with predicted probability of response between 30% and 80%, posterior probabilities did not differ significantly from prior probabilities of response or nonresponse.

### Validation of prediction model

In the validation sample of 176 new completer cases treated with SSRI drugs, the distributions of clinical characteristics and genetic markers did not differ from the derivation sample (Table 1, Table S5). Seven of the top 10 SNPs that showed the strongest association with SSRI treatment response in the derivation sample were significantly associated in the validation sample (Table S6). In this validation cohort, the prior probabilities were 66% for response (116 of 176) and 34% for nonresponse (60 of 176) (Table 1). The HAP-SNP model made predictions for 60% (106/176) of these patients, with 84 predicted responders and 22 predicted nonresponders (Figure 3B). The observed outcomes in these 106 predicted cases were 74 responders and 32 nonresponders (Figure 3B). The overall accuracy of prediction was 85% (90/106; [78%–92%]). Sensitivity and specificity were 96% (71/74; [91%–100%]) and 59% (19/32; [42%–76%]), respectively. This specificity result was not significantly lower than in the derivation sample (*P* = 0.21). Among these 106 cases, the prior probabilities of observed response (70%) and nonresponse (30%) in the absence of genotyping increased to posterior probabilities of 85% (PPV; 71/84; [77%–92%]) and 86% (NPV; 19/22; [72%–100%]), respectively (*P*<0.001 in each case). These PPV and NPV results did not significantly differ from the corresponding values (PPV = 88%, NPV = 85%) in the derivation sample (Fisher's exact test, *P* = 0.66, *P* = 1.00, respectively). Of note, the model predicted 59% (19 of 32) of observed nonresponders in the validation sample with 86% accuracy (Figure 3B).

### Cross-validation of prediction model

In the third phase of this study we tested whether the HAP-SNP model that predicted response to SSRIs also predicted outcomes in patients treated with non-SSRI antidepressants. In an independent sample of 189 protocol completers (Figure 1), we compared response and nonresponse predicted for SSRIs by the HAP-SNP model with observed response and observed nonresponse to non-SSRI antidepressants. The distributions of clinical characteristics and genetic markers did not differ from the derivation sample in this cross-validation sample (Table 1, Table S5). The observed response rate was 60% (114/189). The genetic associations with observed response to SSRI drugs were not replicated for observed response to non-SSRI agents (Table S7). Figure S3 shows a lack of correlation between the association analysis *P* values of groups receiving SSRIs and non-SSRIs (Pearson r = 0.02). No SNP markers in the non-SSRI group reached a significant level of association with response after FDR correction.

Consistent with these gene association differences, the observed responses to non-SSRI drugs differed significantly from the predictions made by the HAP-SNP model that predicted response to SSRIs. The model made predictions for 84 (44%) of the 189 cases in the cross validation sample (61 predicted responders (73%) and 23 predicted nonresponders (27%)). There were 43 observed responders (70.5%) among the 61 predicted responders (PPV), and 11 observed nonresponders (47.8%) among the 23 predicted nonresponders (NPV). These values were significantly different from the corresponding PPV and NPV values in the derivation sample. (Fisher's exact test, *P* = 0.01, *P*<0.01, respectively). Within this group of 84 cases, the observed outcomes were 55 responders (65%) and 29 nonresponders (35%). These are the prior probabilities of response and nonresponse among predicted cases, not significantly different from the full cohort of 189 cases (60% and 40%, respectively).

Text S2 provides additional descriptions of secondary analyses (Supplementary Results), which describes (1) comparisons of the three cohorts in respect of genotypes, clinical characteristics, and plasma drug levels in relation to response status; (2) SNP associations with the secondary outcome of remission; (3) a test of the top 10 SNPs in the response prediction model for possible associations with the diagnosis of major depression – with no significant association being found; (4) details of the polymorphism prediction model that was replaced by the HAP-SNP model; (5) secondary conditional probability analyses in the cross-validation cohort, demonstrating a double dissociation of observed versus expected outcomes: cases predicted by the HAP-SNP model to do poorly with SSRI treatment actually had significantly better observed outcomes with non-SSRI treatment, while cases predicted by the HAP-SNP model to do well with SSRI treatment actually had significantly worse observed outcomes with non-SSRI drugs.

## Discussion

The markers associated with response to SSRI drugs comprised ten SNPs from the *TPH2, SLC6A4, GRIK2,* and *GAD1* genes and six haplotypes from the *TPH2, SLC6A4,* and *GRIK2* genes (Table 2 and Figure 2). Thus, SSRI response was associated with polymorphisms in serotonin, glutamate, and GABA related genes. *TPH2* showed the most significant association with SSRI response. *TPH2* encodes the rate-limiting enzyme of brain serotonin production [28].

### Comparison with previous studies

Our finding of association between *TPH2* and SSRI response is consistent with prior evidence from studies in an animal model and human post mortem neurochemistry [29], [30]. We found associations of SSRI response with 4 SNPs in *TPH2* (rs4760815, rs11179027, rs17110532, and rs17110747). A previous small study found that three SNPs in *TPH2*, rs1843809 and rs1386492 of intron 5, and rs1487276 of intron 8, were associated with drug response after 12 weeks of SSRI treatment (Figure S4) [31]. However, there was no significant association between those three SNPs and SSRI response in the present study.

Another study performed in a European population investigated nine SNPs in the *TPH2* gene, and found two SNPs, rs10879346 and rs1487278, were significantly related to antidepressant response [32]. Additionally, rs2171363 was significant in a Chinese population [33]. We imputed these three SNPs using genotype data, because they were not genotyped in our study. The imputed SNPs showed significant associations with SSRI response (Figure S4). These results from imputed data increase the possibility that the predictive markers suggested in our data will be replicable in other populations.

Our previous studies indicated that two VNTRs in the 2^nd^ intron (*STin2*) and promoter (*5-HTTLPR*) of *SLC6A4* are associated with SSRI response [5], [9]. In this study, we examined the two VNTRs and 12 SNPs in the *SLC6A4* gene, and found both VNTRs and two SNPs were significantly associated with SSRI response (Table 2). The two SNPs, rs2066713 and rs2020942, were located in intron 1 and intron 3, respectively. A previous study reported that these two SNPs have no association with SSRI response at 12 weeks in an ethnically mixed population [31], but another U.S. study reported that rs2066713 showed a trend towards association with SSRI response [34]. Three polymorphisms significantly associated with SSRI response, rs2066713 of intron 1, VNTR of intron 2, and rs2020942 of intron 3, were tightly linked (Table S8). When we constructed a haplotype from 12 SNPs of the *SLC6A4* gene, the haplotype was significantly associated with SSRI response (Figure 2C). It was also reported in a Caucasian population that a haplotype constructed from 21 SNPs of the *SLC6A4* gene was significant [31]. Thus, our results here and previously [5], [9] are consistent with much previous work and with a previous meta-analysis [35] which concluded that the *SLC6A4* gene is an informative genetic marker for SSRI response. Moreover, a recent meta-analysis study [36] that examined Caucasian and Asian populations separately confirmed the importance of ethnicity for interpreting pharmacogenetic studies [37]. This is in contrast to an earlier meta-analysis [38] that disregarded ethnicity and found no overall association of *5-HTTLPR* genotype and responsiveness. Porcelli and colleagues reported in Caucasians that *5-HTTLPR* may be a predictor of antidepressant response, while in Asians it is not. These inconsistencies in the evidence for an association between *5-HTTLPR* and antidepressant response in Asian populations may result also from the established genetic variability within broad Asian ethnic groups. For instance, the genotype distribution of *5-HTTLPR* in Han Chinese is closer to the Caucasian profile than to the Japanese or Korean profile [37].

We did not confirm the claim that the serotonin receptor gene *HTR2A* is associated with SSRI response [8]. We found no association for any of the 28 markers in the *HTR2A* gene in our population (rs7997012, FDR corrected *P* = 0.47). In addition, the original report [8] has not been consistently replicated [12], [39].

We found that both glutamate (*GRIK2*) and GABA (*GAD1*) related genes are associated with SSRI response. Both these abundant neurotransmitters are implicated in mood circuitry. Our result with *GRIK2* might be related to a report claiming *GRIK4* is associated with response to the SSRI citalopram [40]. We found that two SNPs in intron 1 of the *GRIK2* gene (rs543196 and rs572487) and two haplotypes including each SNP were significantly associated with SSRI response, and several neighboring SNPs showed a trend towards association (Table 2 and Figure 2B). The *GRIK2* gene encodes glutamate receptors, which respond to glutamate for excitatory transmission in mood circuits. There are abnormalities in glutamatergic neurotransmission in depressed patients [41], and the glutamate system is influenced by SSRIs [42], [43].

We know of no previous reports that GABA (*GAD1*) related genes are associated with SSRI response. *GAD1* is the key enzyme of the GABA neurotransmitter system. We found that two SNPs in the *GAD1* gene (rs3828275 of intron3 and rs12185692 of 5′-untranslated region) were significantly associated with SSRI response (Table 2). Abnormalities in GABA neurotransmission have been noted in depression [44]. Overall, the genetic profile of our HAP-SNP model for prediction of response to SSRIs is consistent with drug actions involving the neuromodulator serotonin, followed by effects on the mood circuits that employ glutamate and GABA [45].

### Study limitation and strength

A recent meta-analysis identified no individual SNP associations with a genome-wide significance for response to SSRI drugs in depression. That null result includes our own findings [46]. One candidate reason for this apparent non-confirmation may be the ethnic distinctiveness of our population. It is previously reported that response to the SSRI citalopram in African American depressed patients was poorer than in Caucasian Americans and it was suggested that this variance in response may be explained by an allelic frequency difference in rs7997012 of *HTR2A* between the two population samples [8]. Three recent genome-wide association studies [12], [13], [47] failed to identify gene associations with response to antidepressant drugs in depression. These failures underscore the heterogeneity of the clinical depression phenotype, and the complex gene-environment nature of the disorder. In addition, these large, multi-site studies risk incurring methodological problems such as heterogeneity of case material, ethnic heterogeneity, measurement error, and variable recruitment practices [48], [49]. By comparison, strengths of our study design include single site performance by an experienced research team, strictly blinded quality control, ethnic homogeneity, inclusion of only clinically referred cases, clinical diagnoses by experienced psychiatrists in advance of confirmatory research diagnostic interviews [50], outcome assessments in person rather than by telephone, and verification of adequate antidepressant blood levels. We also required that all cases were unexposed to antidepressant drugs in the current episode of depression before enrolment in this study. By these means, heterogeneity and confounding of the case material were controlled, and we succeeded in identifying and validating significant genetic predictors of response with manageable sample sizes.

The prediction model examined *observed* response and nonresponse: without a placebo control group we have no basis to predict specific drug response. The gain of information from the predictive model is substantial, especially in the prediction of nonresponse. For the 16% of completer cases (39/239) that our HAP-SNP model predicts will be nonresponders in the derivation sample (Figure 3A), the relative risk of observed nonresponse is 3.3 in comparison to all other cases, and 6.9 in comparison to the cases whom the model predicts will be responders. In the validation sample, these relative risks are 3.2 and 5.6, respectively (Figure 3B). For comparison, the relative risk of a poor outcome is 1.5 in the 27% of patients receiving clopidogrel who have loss of function polymorphisms in *CYP2C19* [51].

The genetic determinants of observed response to SSRI drugs (Table 2) were not associated with response to non-SSRI antidepressant drugs (Table S7). Thus, these results are consistent with the previous reports [9], [11] that pharmacologically different antidepressants are associated with different genetic determinants of response. A further, indirect, inference is that the significant markers for observed response to SSRI drugs may be unrelated to nonspecific response factors (“placebo effect”) in our patients. However, we should mention that previous antidepressant treatment history in prior episodes of depression might have influenced the clinicians' choice of non-SSRI treatment in the cross-validation sample. We cannot positively rule out this possible confound in this naturalistic study, even though the cross-validation sample closely resembled the SSRI-treated samples on relevant clinical variables (Table 1).

The convergent data from the validation and cross-validation samples suggest that for approximately half the total cases who adhere to treatment, a gene-based recommendation of SSRI or non-SSRI agent as first-line treatment may be possible with 85% confidence, and that this represents a significant improvement over base rates of response and nonresponse in the absence of genotype information for those cases.

The ethnic homogeneity of our sample may be viewed as either a strength or a limitation, and our prediction model needs to be evaluated in other populations. However, the predictive markers suggested in the European [32] and Chinese studies [33] were replicable in our population from imputed data of *TPH2* (Figure S4). Moreover, the ethnic homogeneity of our sample with the appropriate power may overcome the problems of population stratification which can occur in ethnically mixed populations [8]. Additionally, we could not detect any evidence of population stratification between responders and nonresponders in the 1400 genetic markers of our subjects by the Structure 2.2 software [52] and by quantile-quantile plots of the association results (Figure S5 and Figure S6).

Our prediction model does not include clinical variables. Duration of depressive episode was the only clinical or demographic variable that differed between responders and nonresponders, and only in the derivation sample (Table 1). This clinical variable was eliminated when it was found to be nonsignificant in the logistic regression analyses. Thus, while clinical features are somewhat related to antidepressant response, they may not be independently predictive after correction for genomic factors [32].

### Implications

Our HAP-SNP model appears to achieve the goal of gene-based selection of drug class in just over 50% of adherent cases. Though it remains an objective, we do not yet know whether it is realistic to expect significantly better predictive power than 50% in such a complex and heterogeneous disorder as DSM-IV defined major depression. Nevertheless, this extent of genetic prediction is potentially cost-effective [53]. In particular, 59% of the anticipated nonresponders could be identified without the expense and delay associated with a failed trial of SSRIs. In order to evaluate the applicability of genetic predictors in clinical practice, Intent-to-Treat (ITT) analyses and cost analyses will be required. However, ITT is not the appropriate framework for discovery purposes such as this study. Moreover, all potential biomarkers for prediction of antidepressant response in practice settings are destined to be subject to the attrition that we observed (at least 20%), if not much more [54]. While our results need to be confirmed in other populations, and will doubtless be refined with further experience, to the best of our knowledge, no genetic models possessing comparable power have been proposed and validated for the prediction of antidepressant drug class response.

**Web Resources**

dbSNP: http://www.ncbi.nlm.nih.gov/projects/SNP

FESD: http://sysbio.kribb.re.kr:8080/fesd

HapMap: http://hapmap.org

Tagger: http://www.broad.mit.edu/mpg/tagger

## Supporting Information

Figure S1 **Selection of 1502 candidate SNPs from 79 candidate genes.**
(TIF)

Figure S2 **Difference in linkage disequilibrium (LD) structure of TPH2 between responders and nonresponders.** The LD structure is based on the measure of *r^2^*. LD was stronger among responders than among nonresponders. The region including three different haplotype blocks, H2, H3, and a part of H4 in the nonresponder group was observed as a single long haplotype, H2 (12 SNPs) in the responder group.
(TIF)

Figure S3 **Association analysis of SSRI and non-SSRI treated groups.** Association analysis p values (as –log_10_ values) of 1400 polymorphic markers were plotted. No correlation of p values between SSRI treated and non-SSRI treated groups was observed. (A) Association analysis p values between antidepressant response and single-nucleotide polymorphisms (SNPs) in the SSRI treated group are sorted in descending order. The plot of the p values of the SSRI treated group is distributed by continuous curve form and that of the non-SSRI treated group by scattered form. (B) The distribution of high –log_10_ p values on each axis demonstrates that distinct SNPs were associated with response to each class of antidepressant drug.
(TIF)

Figure S4 **Comparison of association analysis results for** ***TPH2*** **with previous studies.** Red filled diamonds indicate genotyped SNPs in this study and red blank diamonds imputed SNPs. Blue filled triangles, blue blank triangles, and dark green crosses indicate association results studied in Tzvetkov et al. 2008, Peters et al. 2004, and Tsai et al. 2009, respectively. The same SNPs between studies are linked by dotted lines. The significant SNPs, rs10879346 and rs1487278, in Tzvetkov et al. 2008 study and rs2171363 in Tsai et al. 2009 study were replicated in our imputation study, suggesting that associations in the current study might be replicable in other populations (See Discussion of Manuscript).
(TIF)

Figure S5 **Population structure in the derivation sample of SSRI treated patients.** Population structure was estimated from 10 000 iterated simulations using the Structure 2.2 software. Red and green circles indicate responders and nonresponders, respectively. We set the number (*K*) of possible sub-populations as three (cluster 1, cluster 2 and others). If there was population stratification, individual circles would be grouped near one of the clusters according to their overall genetic similarity. We did not observe any clear pattern of clustering between responders and nonresponders. No evidence of population stratification between two groups was observed in our sample.
(TIF)

Figure S6 **Quantile-quantile (QQ) plots for association tests of 1400 SNPs.** For each of five genetic modes of (A) genotype, (B) additive, (C) allele, (D) dominant, and (E) recessive, QQ plots of the results of association with selective serotonin reuptake inhibitor (SSRI) response are shown in blue. No overall departures of the observed p values from the expected p values were observed in the QQ plots. Median value of –log_10_ *P* values ranged from 0.24 to 0.34 according to the genetic mode.
(TIF)

Table S1 Candidate genes and 1502 selected SNPs.
(DOCX)

Table S2 Summary of selected SNPs according to SNP selection method.
(DOCX)

Table S3 Associations of significant SNP markers with response for individual SSRI drugs in derivation sample.
(DOCX)

Table S4 Plasma levels of antidepressants for responders and nonresponders.
(DOCX)

Table S5 Distribution of genotypes of the SNPs most strongly associated with response to SSRIs in derivation sample, in validation sample, and in cross-validation sample.
(DOCX)

Table S6 Association analysis results in validation samples of top 10 SNPs significantly associated with SSRI response in derivation samples.
(DOCX)

Table S7 SNPs most strongly associated with response to non-SSRI drugs.
(DOCX)

Table S8 Linkage disequilibrium (LD) between predictive markers.
(DOCX)

Text S1 **Supplementary Methods**: 1. Function-based tagging selection of single-nucleotide polymorphism markers; 2. Genotyping, 3. Plasma drug levels; 4. Power analysis.
(DOCX)

Text S2 **Supplementary Results**: 1. Characteristics of study subjects; 2. SNPs most strongly associated with remission in SSRI treated group; 3. Association with depression diagnosis for top 10 SNPs significantly associated with SSRI response; 4. Polymorphism prediction model for SSRI treatment; 5. Cross validation results with HAP-SNP prediction model.
(DOCX)
