# Supplementary figures and images for: Genetic Prediction of Antidepressant Drug Response and Nonresponse in Korean Patients
Source: PLoS One. 2014 Sep 16;9(9):e107098. doi: 10.1371/journal.pone.0107098 (PMC4166419; doi:10.1371/journal.pone.0107098)

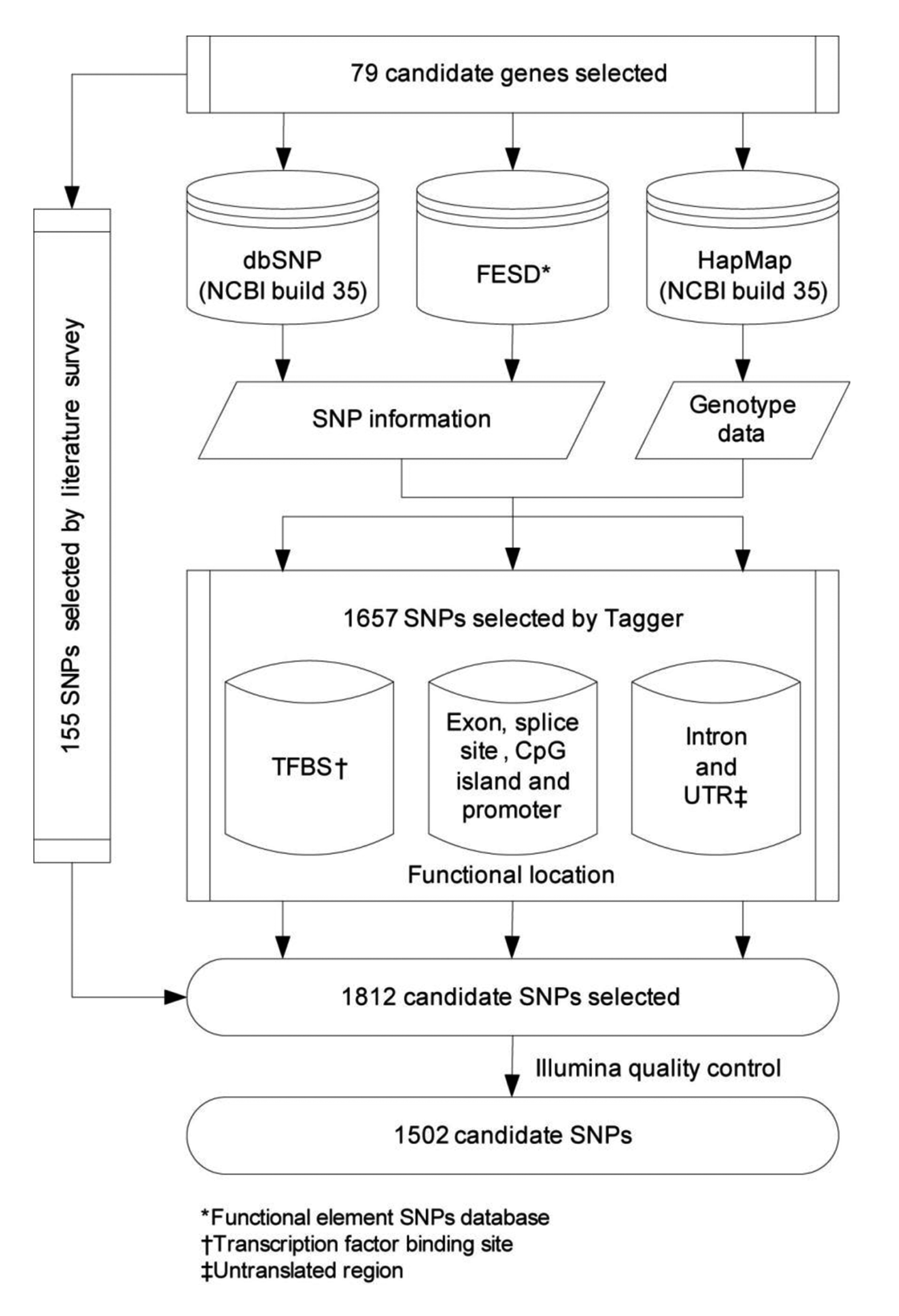

Supplement: Figure S1 — Selection of 1502 candidate SNPs from 79 candidate genes. (TIF) [file pone.0107098.s001.tif]

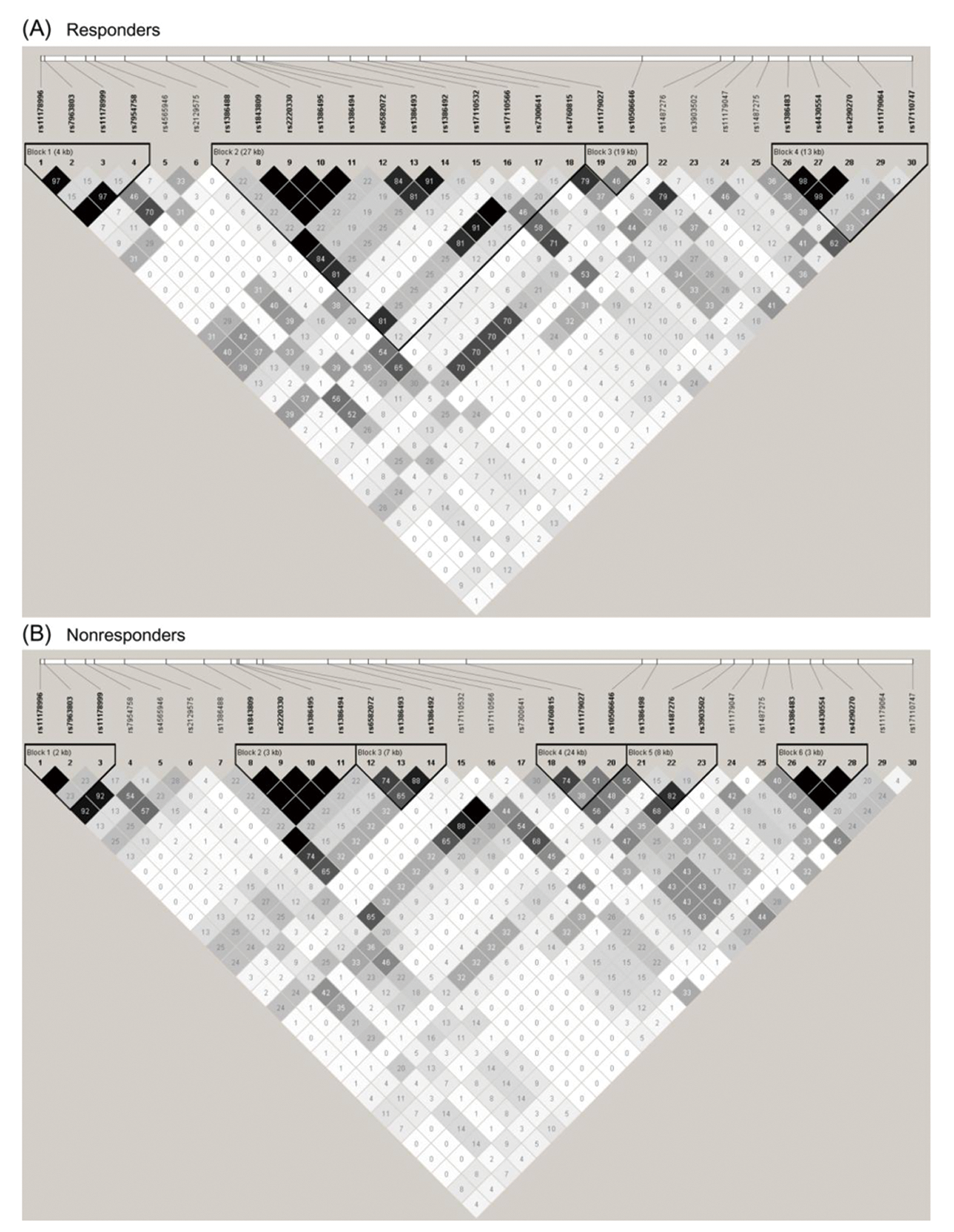

Supplement: Figure S2 — Difference in linkage disequilibrium (LD) structure of TPH2 between responders and nonresponders. The LD structure is based on the measure of r2. LD was stronger among responders than among nonresponders. The region including three different haplotype blocks, H2, H3, and a part of H4 in the nonresponder group was observed as a single long haplotype, H2 (12 SNPs) in the responder group. (TIF) [file pone.0107098.s002.tif]

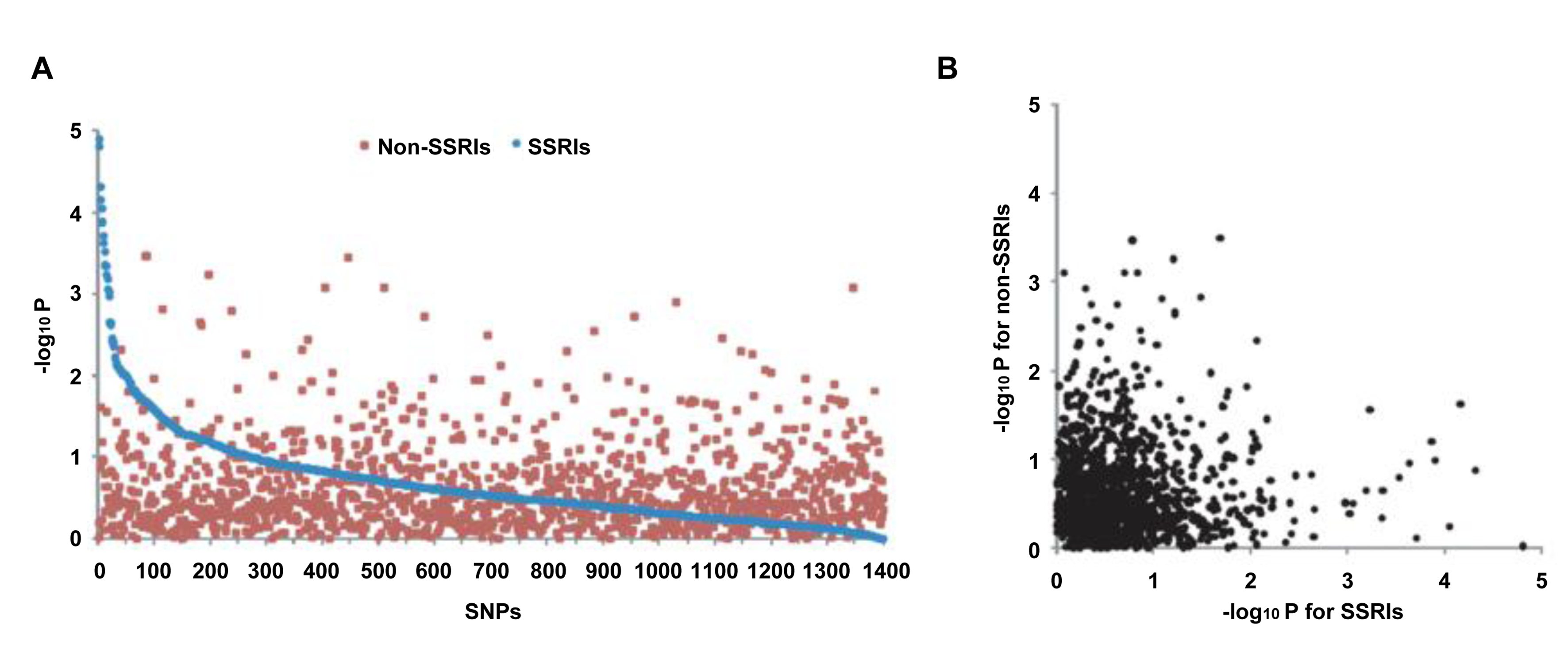

Supplement: Figure S3 — Association analysis of SSRI and non-SSRI treated groups. Association analysis p values (as –log10 values) of 1400 polymorphic markers were plotted. No correlation of p values between SSRI treated and non-SSRI treated groups was observed. (A) Association analysis p values between antidepressant response and single-nucleotide polymorphisms (SNPs) in the SSRI treated group are sorted in descending order. The plot of the p values of the SSRI treated group is distributed by continuous curve form and that of the non-SSRI treated group by scattered form. (B) The distribution of high –log10 p values on each axis demonstrates that distinct SNPs were associated with response to each class of antidepressant drug. (TIF) [file pone.0107098.s003.tif]

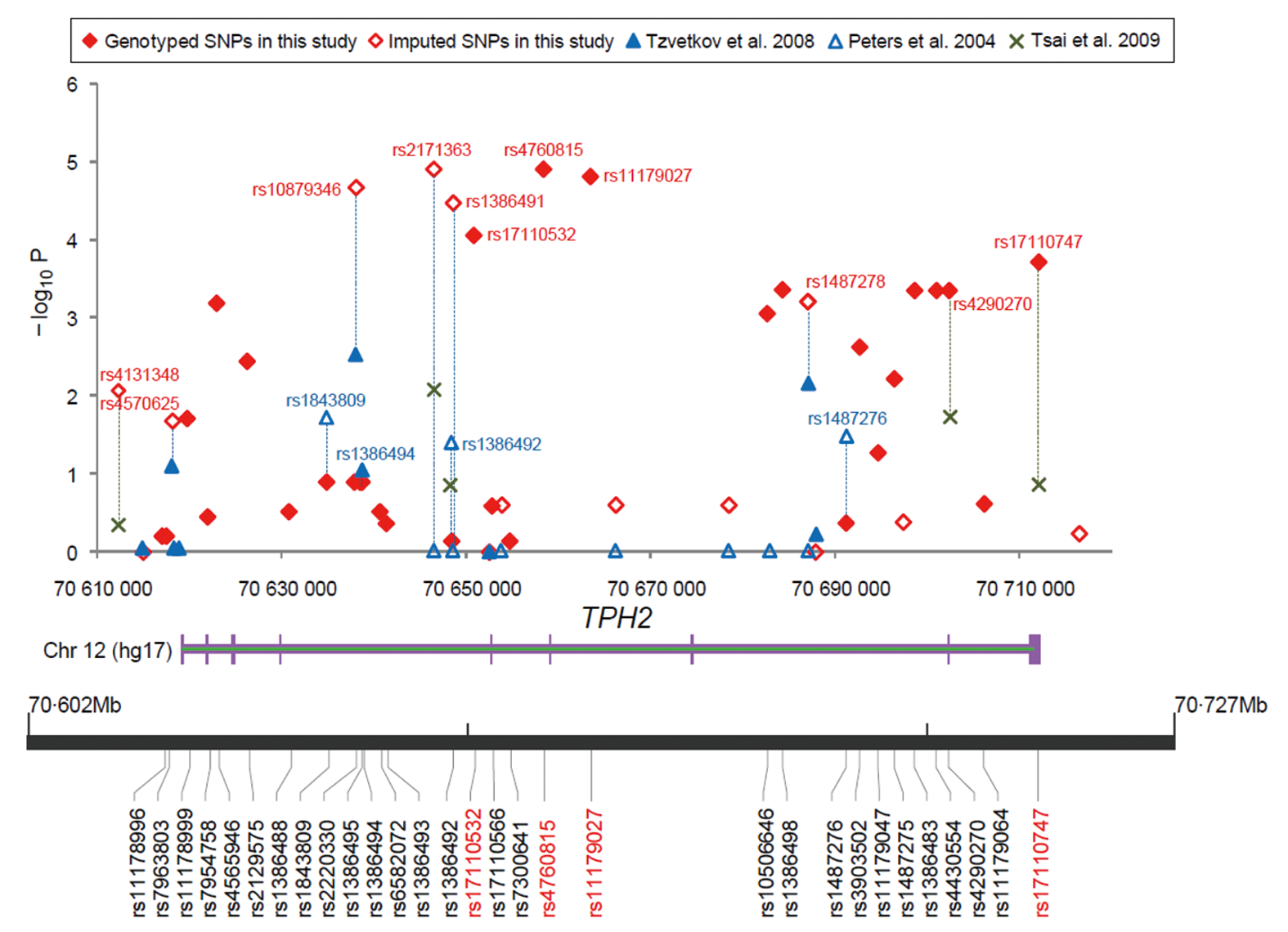

Supplement: Figure S4 — Comparison of association analysis results for TPH2 with previous studies. Red filled diamonds indicate genotyped SNPs in this study and red blank diamonds imputed SNPs. Blue filled triangles, blue blank triangles, and dark green crosses indicate association results studied in Tzvetkov et al. 2008, Peters et al. 2004, and Tsai et al. 2009, respectively. The same SNPs between studies are linked by dotted lines. The significant SNPs, rs10879346 and rs1487278, in Tzvetkov et al. 2008 study and rs2171363 in Tsai et al. 2009 study were replicated in our imputation study, suggesting that associations in the current study might be replicable in other populations (See Discussion of Manuscript). (TIF) [file pone.0107098.s004.tif]

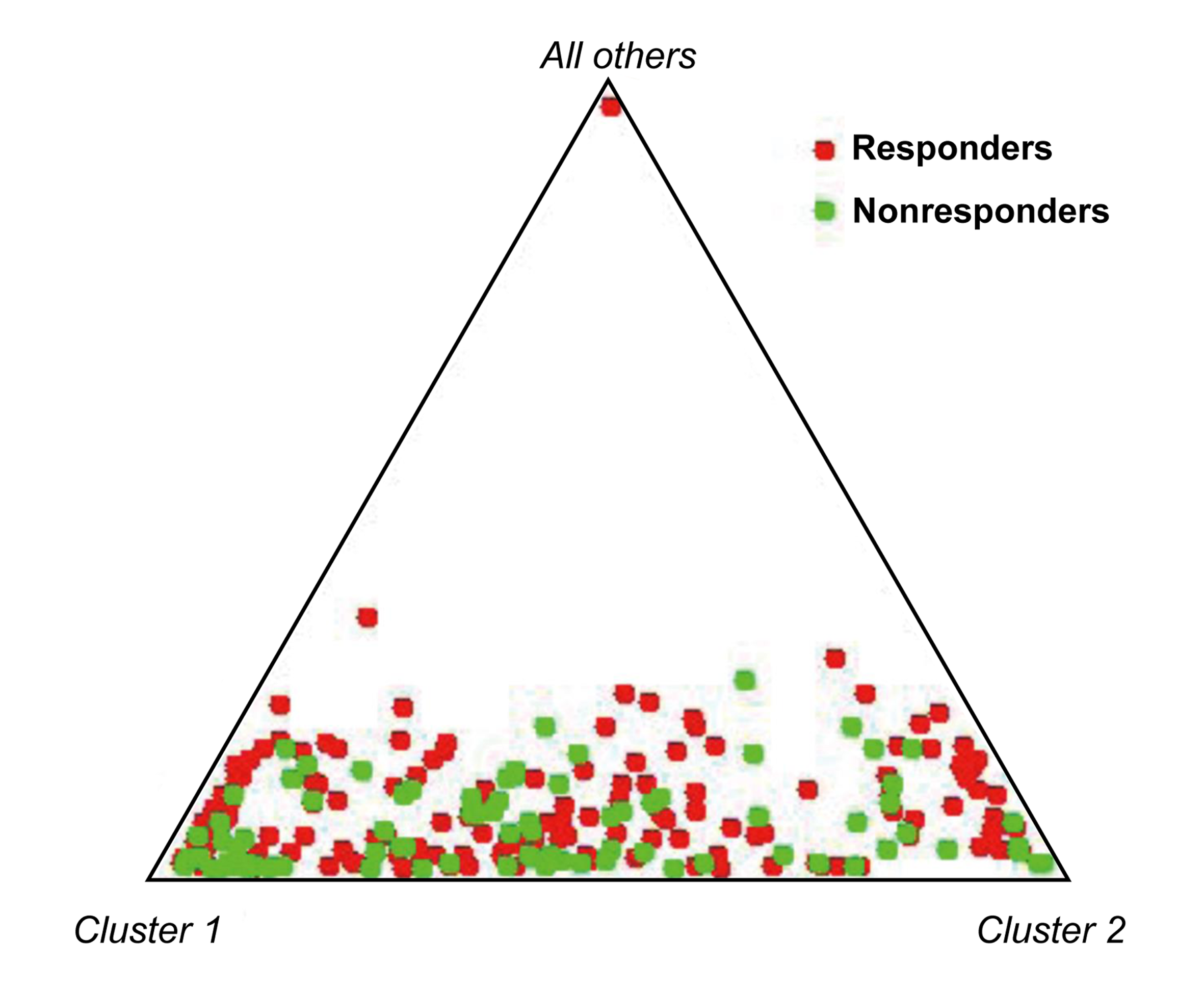

Supplement: Figure S5 — Population structure in the derivation sample of SSRI treated patients. Population structure was estimated from 10 000 iterated simulations using the Structure 2.2 software. Red and green circles indicate responders and nonresponders, respectively. We set the number (K) of possible sub-populations as three (cluster 1, cluster 2 and others). If there was population stratification, individual circles would be grouped near one of the clusters according to their overall genetic similarity. We did not observe any clear pattern of clustering between responders and nonresponders. No evidence of population stratification between two groups was observed in our sample. (TIF) [file pone.0107098.s005.tif]

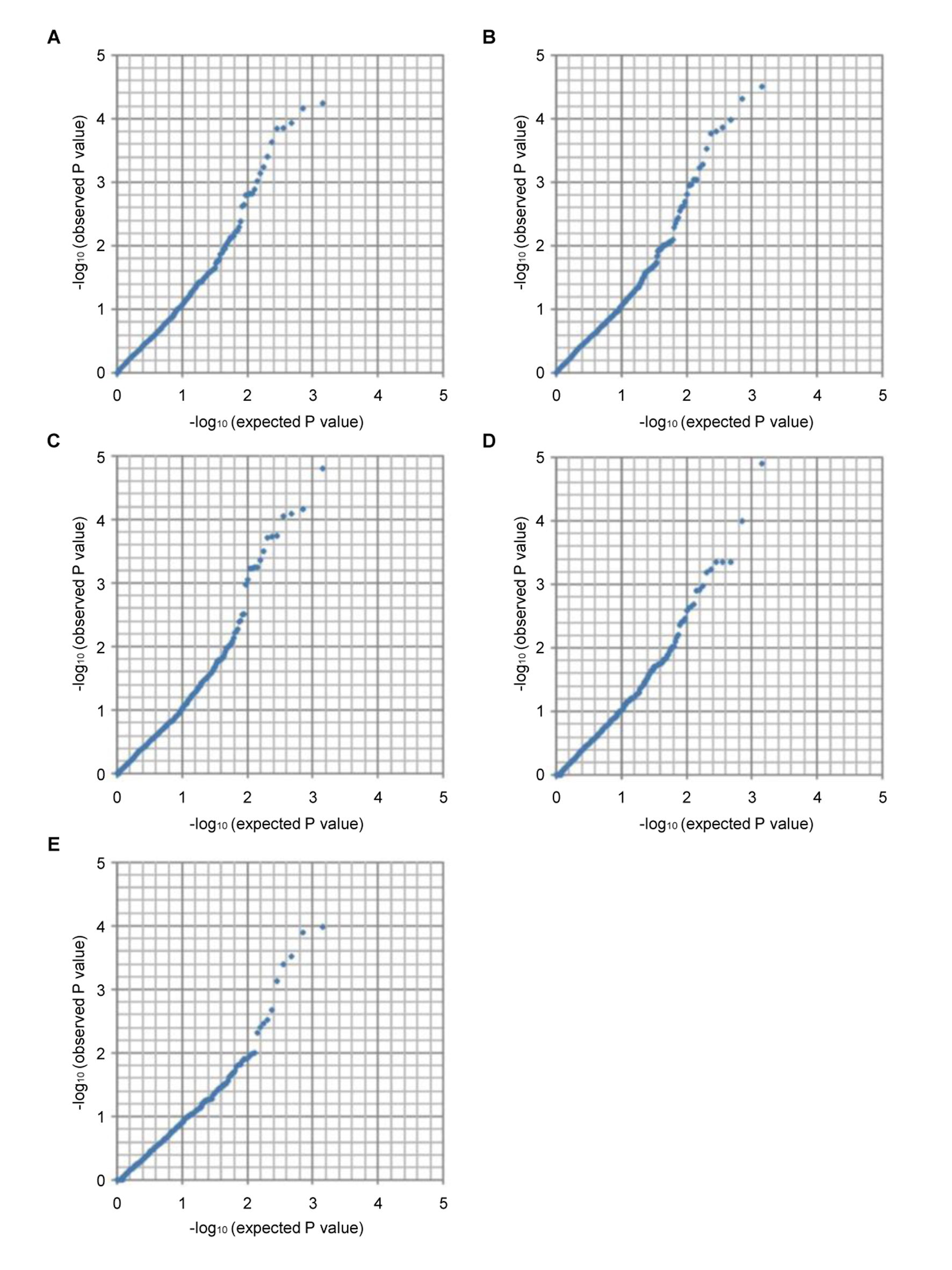

Supplement: Figure S6 — Quantile-quantile (QQ) plots for association tests of 1400 SNPs. For each of five genetic modes of (A) genotype, (B) additive, (C) allele, (D) dominant, and (E) recessive, QQ plots of the results of association with selective serotonin reuptake inhibitor (SSRI) response are shown in blue. No overall departures of the observed p values from the expected p values were observed in the QQ plots. Median value of –log10 P values ranged from 0.24 to 0.34 according to the genetic mode. (TIF) [file pone.0107098.s006.tif]
