# Supplementary material for: Genetic Prediction of Antidepressant Drug Response and Nonresponse in Korean Patients
Source: PLoS One. 2014 Sep 16;9(9):e107098. doi: 10.1371/journal.pone.0107098 (PMC4166419; doi:10.1371/journal.pone.0107098)
Supplement: Table S1 — Candidate genes and 1502 selected SNPs. (DOCX) [file pone.0107098.s007.docx]

**Table S1** Candidate genes and 1502 selected SNPs

| **Class** | **No. of genes** | **Genes (No. of selected SNPs)** | | |
| --- | --- | --- | --- | --- |
| Transporter | 5 | *ABCB1* (23), *SLC1A1* (36), *SLC6A2* (26), *SLC6A3* (7), *SLC6A4* (12) | |  |
| Metabolic enzyme | 16 | *TPH1* (13), *TPH2* (32), *TH* (7), *MAOA* (3), *MAOB* (4), *COMT* (17), *DBH* (13), *PAM* (11), *ACE* (8), *MTHFR* (8), *PNMT* (3), *GAD1* (11), *CPOX* (8), *DAO* (3), *DAOA* (11), *AOC2* (1) | |  |
| Receptor | 58 | Indoleamine | Serotonergic System: *HTR1A* (3), *HTR1B* (6), *HTR1D* (3), *HTR1E* (15), *HTR1F* (2), *HTR2A* (28), *HTR2C* (8), *HTR3B* (8), *HTR5A* (5), *HTR6* (3), *HTR7* (13) |  |
|  |  | Catecholamine | Dopaminergic System: *DRD1* (3), *DRD2* (11), *DRD3* (7), *DRD4* (1)  Noradernergic System: *ADRA1A* (33), *ADRA1B* (9), *ADRA2A* (5), *ADRB1* (2), *ADRB2* (7), *ADRB3* (1) |  |
|  |  | Acetylcholine | *CHRM1* (3), *CHRM2* (22) |  |
|  |  | Glutamate | Ionotropic N-methyl-D-aspartic Acid (NMDA): *GRIN2A* (61), *GRIN2D* (6), *GRIN3A* (47)  Non-NMDA: *GRIA1* (32), *GRIA2* (7), *GRIA3* (54), *GRIA4* (30), *GRIK1* (69), *GRIK2* (83)  Metabotropic NMDA: *GRM1* (37), *GRM3* (18), *GRM4* (37), *GRM5* (72), *GRM7* (221), *GRM8* (47) |  |
|  |  | GABA, Glycine | *GABBR1* (8), *GABRA1* (7), *GABRG2* (16), *GLRB* (5) |  |
|  |  | Neuropeptide | *CCK* (6), *CCKAR* (7), *TAC1* (6), *TACR1* (51), *NPY* (8), *OPRD1* (8), *OPRM1* (11), *OPRS1* (1), *CNR1* (4), *AVPR2* (4) |  |
|  |  | Channel | *KCNA1* (2), *KCNH2* (6), *KCNJ3* (41), *KCNK2* (16), *KCNN1* (10), *KCNN2* (9) |  |
