# Supplementary material for: Genetic Prediction of Antidepressant Drug Response and Nonresponse in Korean Patients
Source: PLoS One. 2014 Sep 16;9(9):e107098. doi: 10.1371/journal.pone.0107098 (PMC4166419; doi:10.1371/journal.pone.0107098)
Supplement: Table S2 — Summary of selected SNPs according to SNP selection method. (DOCX) [file pone.0107098.s008.docx]

**Table S2** Summary of selected SNPs according to SNP selection method

| **Source** | **No. of SNPs in dbSNP** | **No. of SNPs in HapMap** | **Selecting condition** | **No. of finally selected SNPs*** | **Tagging efficiency†** |
| --- | --- | --- | --- | --- | --- |
| Literature survey | 155 |  | No processing | 155 | No Tagging |
| *SLC6A4* |  | 337 | Tagging with MAF≥0.05 and *r^2^*=1.00 | 54 | 16.0% (54/337) |
| TFBS | 54 | 16 | Excluding SNPs with  MAF<0.05 | 48 | No Tagging |
| Exon, splice site, CpG island and promoter region | 1303 | 447 | Tagging with MAF≥0.05 and *r^2^*≥0.90 | 146 (40) | 32.7% (146/447) |
| Intron and UTR | 31 731 | 14 582 | Tagging with MAF≥0.10 and *r^2^*≥0.80 | 1458 (9) | 10.0% (1458/14 582) |
| Total selected SNPs |  |  |  | 1812 |  |

Abbreviations: MAF, minor allele frequency; TFBS, transcription factor binding site; UTR, untranslated region.

***** The number in parenthesis indicates the number of SNPs that are also included in other source categories.

† Tagging efficiency is defined as the proportion of the tagged SNPs among the raw SNPs before tagging.
