# Supplementary material for: Genetic Prediction of Antidepressant Drug Response and Nonresponse in Korean Patients
Source: PLoS One. 2014 Sep 16;9(9):e107098. doi: 10.1371/journal.pone.0107098 (PMC4166419; doi:10.1371/journal.pone.0107098)
Supplement: Table S3 — Associations of significant SNP markers with response for individual SSRI drugs in derivation sample. (DOCX) [file pone.0107098.s009.docx]

**Table S3** Associations of significant SNP markers with response for individual SSRI drugs in derivation sample

|  |  | **Fluoxetine** | | **Paroxetine** | | **Sertraline** | |  |
| --- | --- | --- | --- | --- | --- | --- | --- | --- |
|  |  | **Response (%)** | **Response**  **/total (N)** | **Response (%)** | **Response**  **/total (N)** | **Response (%)** | **Response**  **/total (N)** | ***P* value** |
| rs11179027* | CC | 85.0 | 17/20 | 73.3 | 11/15 | 79.2 | 19/24 | (0.69)  1.00 |
|  | CG | 68.1 | 32/47 | 67.9 | 19/28 | 70.6 | 24/34 |  |
|  | GG | 51.4 | 19/37 | 46.2 | 6/13 | 33.3 | 7/21 |  |
| rs543196* | CC | 78.9 | 30/38 | 100.0 | 15/15 | 74.1 | 20/27 | (0.10)  0.86 |
|  | TC | 58.7 | 27/46 | 60.7 | 17/28 | 59.1 | 26/44 |  |
|  | TT | 55.0 | 11/20 | 30.8 | 4/13 | 50.0 | 4/8 |  |
| rs3828275* | AA | 72.7 | 8/11 | 100.0 | 2/2 | 81.8 | 9/11 | (0.86)  1.00 |
|  | AG | 51.3 | 20/39 | 44.4 | 12/27 | 48.6 | 17/35 |  |
|  | GG | 74.1 | 40/54 | 81.5 | 22/27 | 72.7 | 24/33 |  |
| rs17110532* | CC | 83.3 | 10/12 | 88.9 | 8/9 | 85.7 | 12/14 | (0.58)  1.00 |
|  | TC | 69.2 | 27/39 | 66.7 | 16/24 | 71.4 | 25/35 |  |
|  | TT | 58.5 | 31/53 | 52.2 | 12/23 | 43.4 | 13/30 |  |
| rs2066713* | CC | 73.3 | 66/90 | 66.7 | 32/48 | 66.2 | 45/68 | (0.10)  0.91 |
|  | TC | 15.4 | 2/13 | 42.9 | 3/7 | 45.5 | 5/11 |  |
|  | TT | 0.0 | 0/1 | 100.0 | 1/1 |  | 0/0 |  |
| rs572487* | AA | 57.7 | 15/26 | 33.3 | 5/15 | 50.0 | 6/12 | (0.11)  0.98 |
|  | AG | 58.7 | 27/46 | 65.5 | 19/29 | 61.4 | 27/44 |  |
|  | GG | 81.3 | 26/32 | 100.0 | 12/12 | 73.9 | 17/23 |  |
| rs12185692* | AA | 80.0 | 8/10 | 100.0 | 3/3 | 80.0 | 8/10 | (0.51)  1.00 |
|  | AC | 53.8 | 21/39 | 40.7 | 11/27 | 52.6 | 20/38 |  |
|  | CC | 70.9 | 39/55 | 84.6 | 22/26 | 71.0 | 22/31 |  |
| rs2020942* | AA | 0.0 | 0/2 | 100.0 | 1/1 |  | 0/0 | (0.33)  1.00 |
|  | AG | 33.3 | 5/15 | 37.5 | 3/8 | 46.2 | 6/13 |  |
|  | GG | 72.4 | 63/87 | 68.1 | 32/47 | 66.7 | 44/66 |  |
| rs4760815* | AA | 50.0 | 16/32 | 37.5 | 3/8 | 18.8 | 3/16 | (0.16)  1.00 |
|  | AT | 71.1 | 32/45 | 69.0 | 20/29 | 72.2 | 26/36 |  |
|  | TT | 74.1 | 20/27 | 68.4 | 13/19 | 77.8 | 21/27 |  |
| Total† |  | 65.4 | 68/104 | 64.3 | 36/56 | 63.3 | 50/79 | 0.98 |

Abbreviation: SSRI, selective serotonin reuptake inhibitor.

* (Uncorrected) and corrected by Bonferroni’s correction from the results of the test for the interaction effect of each SNP and drug on response using multiple logistic regression.

† Fisher’s exact test**.**
