# Supplementary material for: Genetic Prediction of Antidepressant Drug Response and Nonresponse in Korean Patients
Source: PLoS One. 2014 Sep 16;9(9):e107098. doi: 10.1371/journal.pone.0107098 (PMC4166419; doi:10.1371/journal.pone.0107098)
Supplement: Table S4 — Plasma levels of antidepressants for responders and nonresponders. (DOCX) [file pone.0107098.s010.docx]

**Table S4** Plasma levels of antidepressants for responders and nonresponders

| **Sample** | **Concentration for** | **Responders**  **(ng/mL)**  **Mean±SD** | **Nonresponders**  **(ng/mL)**  **Mean±SD** | | ***P* value*** |
| --- | --- | --- | --- | --- | --- |
| Derivation sample | Fluoxetine/Norfluoxetine | 572±214 | 553±286 | 0.28 | |
|  | Paroxetine | 75±44 | 80±38 | 0.32 | |
|  | Sertraline | 73±30 | 70±27 | 0.82 | |
| Validation  sample | Fluoxetine/Norfluoxetine | 563±235 | 545±235 | 1.00 | |
|  | Paroxetine | 73±35 | 70±37 | 0.66 | |
|  | Sertraline | 68±25 | 71±20 | 0.66 | |
| Cross-validation sample | Milnacipran | 90±32 | 84±20 | 0.86 | |
|  | Venlafaxine/  O-desmethylvenlafaxine | 799±207 | 773±191 | 0.82 | |
|  | Nortriptyline | 117±22 | 121±25 | 0.46 | |
|  | Mirtazapine | 54±29 | 58±32 | 0.62 | |

Abbreviation: SSRI, selective serotonin reuptake inhibitor.

The recommended therapeutic concentration ranges for fluoxetine/norfluoxetine, paroxetine, sertraline, nortriptyline, and mirtazapine plasma concentration are 200–700 ng/ml, 20–200 ng/ml, 30–200 ng/ml, 50–150 ng/ml, and 5–100 ng/ml, respectively [8].

* Mann-Whitney test.
