# Supplementary material for: Genetic Prediction of Antidepressant Drug Response and Nonresponse in Korean Patients
Source: PLoS One. 2014 Sep 16;9(9):e107098. doi: 10.1371/journal.pone.0107098 (PMC4166419; doi:10.1371/journal.pone.0107098)
Supplement: Table S5 — Distribution of genotypes of the SNPs most strongly associated with response to SSRIs in derivation sample, in validation sample, and in cross-validation sample. (DOCX) [file pone.0107098.s011.docx]

**Table S5** Distribution of genotypes of the SNPs most strongly associated with response to SSRIs in derivation sample, in validation sample, and in cross-validation sample

| **SNP (Genotypes)** | **Derivation sample** | **Validation**  **sample** | **Cross-validation**  **sample** | ***P** value** |
| --- | --- | --- | --- | --- |
| rs4760815 (AA/AT/TT) (%) | 23.4/46.1/30.5 | 27.3/43.7/29.0 | 30.7/47.6/21.7 | 0.22 |
| rs11179027 (CC/CG/GG) (%) | 24.7/45.6/29.7 | 23.4/44.0/32.6 | 17.5/45.5/37.0 | 0.33 |
| rs543196 (CC/TC/TT) (%) | 33.5/49.4/17.1 | 37.5/43.2/19.3 | 31.2/45.0/23.8 | 0.36 |
| rs3828275 (AA/AG/GG) (%) | 10.0/42.3/47.7 | 10.8/42.0/47.2 | 7.4/44.4/48.2 | 0.84 |
| rs2066713 (CC/TC/TT) (%) | 86.2/13.0/0.8 | 88.1/11.3/0.6 | 85.2/14.8/0.0 | 0.72 |
| rs572487 (AA/AG/GG) (%) | 22.2/49.8/28.0 | 24.4/43.2/32.4 | 25.9/48.2/25.9 | 0.56 |
| rs17110532 (CC/TC/TT) (%) | 14.6/41.0/44.4 | 13.6/40.9/45.5 | 9.0/43.9/47.1 | 0.49 |
| rs12185692 (AA/AC/CC) (%) | 9.2/43.7/47.1 | 11.8/40.8/47.4 | 6.9/46.0/47.1 | 0.57 |
| rs2020942 (AA/AG/GG) (%) | 1.3/15.0/83.7 | 1.1/13.1/85.8 | 0.0/19.1/80.9 | 0.29 |
| rs17110747 (AA/AG/GG) (%) | 9.6/32.7/57.7 | 9.1/35.4/55.5 | 6.4/36.5/57.1 | 0.72 |

Abbreviation: SSRI, selective serotonin reuptake inhibitor.

* Fisher’s exact test without correction.
