# Supplementary material for: Genetic Prediction of Antidepressant Drug Response and Nonresponse in Korean Patients
Source: PLoS One. 2014 Sep 16;9(9):e107098. doi: 10.1371/journal.pone.0107098 (PMC4166419; doi:10.1371/journal.pone.0107098)
Supplement: Table S6 — Association analysis results in validation samples of top 10 SNPs significantly associated with SSRI response in derivation samples. (DOCX) [file pone.0107098.s012.docx]

**Table S6** Association analysis results in validation samples of top 10 SNPs significantly associated with SSRI response in derivation samples

| **Gene** | **Chromosome** | **Position**† | **SNP** | ***P* value** | **Genetic mode**‡ |
| --- | --- | --- | --- | --- | --- |
| *TPH2* | 12 | 70658496 | rs4760815 | <0.001* | dominant |
| *TPH2* | 12 | 70663579 | rs11179027 | 0.001** | additive |
| *GRIK2* | 6 | 102158042 | rs543196 | 0.018** | additive |
| *GAD1* | 2 | 171390986 | rs3828275 | 0.002* | genotype |
| *TPH2* | 12 | 70650935 | rs17110532 | 0.003** | additive |
| *SLC6A4* | 17 | 25575791 | rs2066713 | 0.080* | recessive |
| *GRIK2* | 6 | 102157181 | rs572487 | 0.063** | additive |
| *TPH2* | 12 | 70712221 | rs17110747 | <0.001* | allele |
| *GAD1* | 2 | 171379072 | rs12185692 | 0.014* | genotype |
| *SLC6A4* | 17 | 25571040 | rs2020942 | 0.123** | additive |

Abbreviations: SSRI, selective serotonin reuptake inhibitor.

*Fisher’s exact test without correction.

**Cochran-Armitage test without correction

†Genomic position (NCBI Build 36).

‡Genetic mode selected in derivation set
