# Supplementary material for: Genetic Prediction of Antidepressant Drug Response and Nonresponse in Korean Patients
Source: PLoS One. 2014 Sep 16;9(9):e107098. doi: 10.1371/journal.pone.0107098 (PMC4166419; doi:10.1371/journal.pone.0107098)
Supplement: Table S7 — SNPs most strongly associated with response to non-SSRI drugs. (DOCX) [file pone.0107098.s013.docx]

**Table S7 S**NPs most strongly associated with response to Non-SSRI drugs

| **Gene** | **Chromosome** | **Position*** | **SNP** | ***P* value†** | ***P* value by controlling FDR** | **Genetic Mode** |
| --- | --- | --- | --- | --- | --- | --- |
| *NPY* | 7 | 24291284 | rs16141 | 3.4×10^–4^ | 0.47 | Dominant |
| *NPY* | 7 | 24291534 | rs9785023 | 3.4×10^–4^ | 0.47 | Dominant |
| *GRIA3* | X | 122418752 | rs2851733 | 3.5×10^–4^ | 0.16 | Allele |
| *KCNJ3* | 2 | 155319639 | rs2349438 | 5.7×10^–4^ | 0.20 | Recessive |
| *CCK* | 3 | 42274741 | rs3774396 | 8.1×10^–4^ | 0.23 | Genotype |
| *TACR1* | 2 | 75264565 | rs2160654 | 8.1×10^–4^ | 0.19 | Allele |
| *GRIA3* | X | 122367886 | rs682478 | 8.2×10^–4^ | 0.16 | Allele |
| *TACR1* | 2 | 75271666 | rs3771861 | 1.2×10^–3^ | 0.21 | Additive |
| *NPY* | 7 | 24289935 | rs16147 | 1.5×10^–3^ | 0.24 | Dominant |
| *GRM1* | 6 | 146754791 | rs362853 | 1.6×10^–3^ | 0.23 | Recessive |

Abbreviations: SSRI, selective serotonin reuptake inhibitor; FDR, false discovery rate.

* Genomic position (NCBI Build 36).

† Fisher’s exact test.
