# Supplementary material for: Genetic Prediction of Antidepressant Drug Response and Nonresponse in Korean Patients
Source: PLoS One. 2014 Sep 16;9(9):e107098. doi: 10.1371/journal.pone.0107098 (PMC4166419; doi:10.1371/journal.pone.0107098)
Supplement: Table S8 — Linkage disequilibrium (LD) between predictive markers. (DOCX) [file pone.0107098.s014.docx]

**Table S8** Linkage Disequilibrium (LD) between predictive markers

| **Gene** | **SNP 1** | **SNP 2** | ***r^2^*** |
| --- | --- | --- | --- |
| *TPH2* | rs4760815 | rs17110532 | 0.47 |
|  | rs4760815 | rs11179027 | 0.78 |
|  | rs17110532 | rs11179027 | 0.58 |
| *GRIK2* | rs543196 | rs572487 | 0.81 |
| *SLC6A4* | *5-HTTLPR* | *STin2* | 0.05 |
|  | *5-HTTLPR* | rs2020942 | 0.06 |
|  | *5-HTTLPR* | rs2066713 | 0.06 |
|  | *STin2* | rs2020942 | 0.97 |
|  | *STin2* | rs2066713 | 0.80 |
|  | rs2020942 | rs2066713 | 0.82 |
| *GAD1* | rs3828275 | rs12185692 | 0.88 |

* For measuring LD, *r^2^* was calculated using Haploview v4.1.
