# Supplementary material for: Genetic Prediction of Antidepressant Drug Response and Nonresponse in Korean Patients
Source: PLoS One. 2014 Sep 16;9(9):e107098. doi: 10.1371/journal.pone.0107098 (PMC4166419; doi:10.1371/journal.pone.0107098)
Supplement: Text S1 — Supplementary Methods: 1. Function-based tagging selection of single-nucleotide polymorphism markers; 2. Genotyping, 3. Plasma drug levels; 4. Power analysis. (DOCX) [file pone.0107098.s015.docx]

**Supplementary Methods**

**1. Function-based tagging selection of single-nucleotide polymorphism (SNP) markers**

To cover genomic regions of the candidate genes effectively, we collected SNP information from dbSNP (http://www.ncbi.nlm.nih.gov/projects/SNP) and the functional element SNPs database (FESD, http://sysbio.kribb.re.kr:8080/fesd) [1]. We classified the entire set of SNPs into three categories according to the potential functional importance of each SNP in its location (Figure S1).

The first category is a subset of SNPs located in transcription factor binding sites (TFBS) of genes. Here, SNPs of a minor allele frequency (MAF) less than 0.05 were excluded. These were not tagged. The second category is a subset of SNPs located in exons, splice sites, CpG islands, and promoter regions. These were tagged by Tagger (http://www.broad.mit.edu/mpg/tagger) [2] with the condition of a MAF≥0.05 and of *r^2^*≥0.90. The third category is a subset of SNPs located in introns and untranslated regions (UTRs). These were tagged with the condition of a MAF≥0.10 and of *r^2^*≥0.80. Because the *SLC6A4* gene (serotonin transporter, 5-hydroxytryptamine transporter, 5-HTT) was found to be significant in our previous studies [3,4], it was tagged with a strict tagging condition of a MAF≥0.05 and of *r^2^*=1.00 to select more SNPs. The tagging efficiency of *SLC6A4* was 16.0% whereas the tagging efficiency of other genes was on average 10.7% (1604/15 029). A higher proportion of SNPs were tagged in the second category than the third category (tagging efficiency: 32.7% *vs*. 10.0%).

The genotype data used for tagging were the SNP data of 90 unrelated Asian individuals comprising 45 Han Chinese in Beijing (HCB) and 45 Japanese in Tokyo (JPT) samples downloaded from the HapMap data repository (NCBI build 35, http://www.hapmap.org).

**2. Genotyping**

Genomic DNA was extracted from whole blood using Wizard Genomic DNA Purification kits (Promega Inc. Madison, WI). Illumina GoldenGate genotyping assays were used for genotyping 1502 SNPs.

Genotype results of thirty four duplicate samples showed high genotype concordance rate (99.94%). For quality control, we discarded 2 samples with a call rate of less than 99%. Sixty seven SNPs with a call rate of less than 95% and 35 SNPs with a MAF of less than 5% were excluded from the 1502 genotyped SNPs. Finally, 1400 SNPs were prepared.

**3. Plasma drug levels**

Plasma levels of antidepressant drugs were quantified through liquid or gas chromatography tandem mass spectrometry according to previous methods [5-9]. Results and reference ranges are shown in Table S4.

**4. Power analysis**

For SSRI treated patients, we assumed 200 of the 1400 SNPs are associated with response based on false discovery rate (FDR) control, and that the maximum ratio of the number of patients with favorable genotypes to non-favorable genotypes is 5.0. With the significance level of 5%, 108 patients are required for power of 0.9. Assuming half of the patients meet these genotype prediction criteria, 216 patients are needed. A validation sample of 176 patients was required to allow a 95% confidence interval of 5 percentage points either side of the true accuracy assuming the estimated accuracy to be 87%.

**References**

1. Kang HJ, Choi KO, Kim BD, Kim S, Kim YJ (2005) FESD: a Functional Element SNPs Database in human. Nucleic Acids Res 33: D518-522.

2. de Bakker PI, Yelensky R, Pe'er I, Gabriel SB, Daly MJ, et al. (2005) Efficiency and power in genetic association studies. Nat Genet 37: 1217-1223.

3. Kim DK, Lim SW, Lee S, Sohn SE, Kim S, et al. (2000) Serotonin transporter gene polymorphism and antidepressant response. Neuroreport 11: 215-219.

4. Kim H, Lim SW, Kim S, Kim JW, Chang YH, et al. (2006) Monoamine transporter gene polymorphisms and antidepressant response in koreans with late-life depression. JAMA 296: 1609-1618.

5. Gex-Fabry M, Balant-Gorgia AE, Balant LP, Rudaz S, Veuthey JL, et al. (2004) Time course of clinical response to venlafaxine: relevance of plasma level and chirality. Eur J Clin Pharmacol 59: 883-891.

6. Higuchi H, Yoshida K, Takahashi H, Naito S, Kamata M, et al. (2003) Milnacipran plasma levels and antidepressant response in Japanese major depressive patients. Hum Psychopharmacol 18: 255-259.

7. Kollroser M, Schober C (2002) Simultaneous determination of seven tricyclic antidepressant drugs in human plasma by direct-injection HPLC-APCI-MS-MS with an ion trap detector. Ther Drug Monit 24: 537-544.

8. Orsulak PJ, Liu PK, Akers LC (2001) Antidepressant drugs. In: Shaw L, editor. The Clinical Toxicology Laboratory. Washington, DC: AACC Press.

9. Tournel G, Houdret N, Hedouin V, Deveau M, Gosset D, et al. (2001) High-performance liquid chromatographic method to screen and quantitate seven selective serotonin reuptake inhibitors in human serum. J Chromatogr B Biomed Sci Appl 761: 147-158.
