# Supplementary material for: Genetic Prediction of Antidepressant Drug Response and Nonresponse in Korean Patients
Source: PLoS One. 2014 Sep 16;9(9):e107098. doi: 10.1371/journal.pone.0107098 (PMC4166419; doi:10.1371/journal.pone.0107098)
Supplement: Text S2 — Supplementary Results: 1. Characteristics of study subjects; 2. SNPs most strongly associated with remission in SSRI treated group; 3. Association with depression diagnosis for top 10 SNPs significantly associated with SSRI response; 4. Polymorphism prediction model for SSRI treatment; 5. Cross validation results with HAP-SNP prediction model. (DOCX) [file pone.0107098.s016.docx]

**Supplementary Results**

**1. Characteristics of study subjects**

In the derivation sample, genotype distributions for each significant polymorphic marker did not differ significantly by drug within the SSRI group (*P* > 0.05). Genotypes were unrelated to drop-out status or UKU side effect score [10]. There was no difference among the SSRI drugs (fluoxetine, paroxetine, and sertraline) in the associations between each significant SNP marker and treatment response (Table S3). The plasma levels of SSRIs for responders and nonresponders were not significantly different (Table S4).

The distributions of clinical characteristics (Figure 1 and Table 1) and genomic markers of 189 patients who received a non-SSRI antidepressant (cross-validation sample) were not different from those of the derivation sample. The choice of drug in the non-SSRI group had no effect on response rate (*P* = 0.35). The plasma levels of non-SSRIs for responders and nonresponders were not significantly different (Table S4).

**2. SNPs most strongly associated with remission in SSRI treated group**

In the SSRI treated derivation sample, the top ten SNPs except two SNPs of *GAD1* also showed association with remission status (not significant after FDR correction, Table S9).

**3. Association with depression diagnosis for top 10 SNPs significantly associated with SSRI response**

We additionally conducted an association analysis comparing the 239 SSRI treated patients of the development sample and 498 normal controls to examine whether these top ten SNPs are related to diagnosis of depression. We could perform association analyses by imputing hundreds of untyped SNPs in normal volunteers of unrelated Korean ancestry comprising 243 male and 255 female subjects. When we tested 325 genotyped and 734 imputed SNPs using the IMPUTE software [11] among the 1502 SNPs, three SNPs, rs4760815, rs11179027, and rs12185692, could not be imputed due to their absence in the HapMap data. None of the remaining seven significant markers showed significant association with the diagnosis (Table S10).

**4. Polymorphism prediction model for SSRI treatment**

In the polymorphism model, ten SNPs which were significantly associated with response and two VNTR markers (*5-HTTLPR* and *STin2*) were considered as predictors. For prediction modeling, among three SNPs with allele genetic mode, we used the genetic mode with the second highest significance for two SNPs (rs11179027, rs17110532) because we could not assign individuals having heterozygous alleles to either of one allele or the other in the model, and rs17110747 was excluded because it showed significance only in the allele mode (Table 2). One VNTR marker (*STin2*) was also excluded due to high linkage disequilibrium (LD) with rs2020942 in this model (*r*^2^=0.97) (Table S8). The polymorphism model finally included rs4760815 of *TPH2* (*P* < 0.001), rs543196 of *GRIK2* (*P* < 0.001), rs3828275 of *GAD1* (*P* < 0.001), rs2066713 of *SLC6A4* (*P* = 0.001), and *5-HTTLPR* (*P* = 0.01) (Table S11), and showed an AUC of 0.81.

The polymorphism model made predictions for 46% of the patients who received SSRI treatment (110 of 239). Seventy eight patients were predicted to be responders, and 32 patients were predicted to be nonresponders. The observed outcomes in these 110 cases were 74 responders and 36 nonresponders (observed response rate 67%). For these 110 cases, the polymorphism model correctly predicted 70 of 74 observed responders (sensitivity 95%; 95% confidence interval [CI], 90%–99%) and 28 of 36 observed nonresponders (specificity 78%; [64%–92%]). The PPV was 90% (70 of 78; [83%–97%]) and the NPV was 88% (28 of 32; [77%–99%]). The overall accuracy or efficiency of prediction by the model was 89% (98 of 110; [83%–95%]). The prior probability of response in the absence of genotyping (67%) increased to a posterior probability of 90% when the model predicted response. The prior probability of nonresponse in the absence of genotyping (33%) increased to a posterior probability of 88% when the model predicted nonresponse. As stated in the manuscript, this polymorphism model was outperformed by the HAP-SNP model.

**5. Cross-validation results with HAP-SNP prediction model**

The HAP-SNP predictive model for response to SSRIs did not predict response to non-SSRI drugs. The model identified 44% (84/189) of the cross validation sample as likely responders (61) or nonresponders (23). The observed responses of these cases to non-SSRI treatment differed significantly from the response expected if they had received SSRI drugs instead. To be conservative, we also applied the PPV (0.85) and the NPV (0.86) obtained in the validation sample in order to estimate the number of expected responders and nonresponders (as distinct from predicted responders and nonresponders) in these two groups. We observed a double dissociation of observed versus predicted outcomes. The expected outcomes for the 23 predicted nonresponders to SSRI treatment were 3 responders, and 20 nonresponders. The observed outcomes of these patients with non-SSRI treatment were 12 responders, and 11 nonresponders (Goodness of Fit Chi-square=27.69, df=1, *P* < 0.0001). Similarly, the expected outcomes for the 61 cases predicted to be responders to SSRI treatment were 52 responders, and nine nonresponders. The observed outcomes of these patients with non-SSRI treatment were 43 responders, and 18 nonresponders (Goodness of Fit Chi-square=9.42, df=1, *P=* 0.0021). Thus, cases predicted by the HAP-SNP model to do poorly with SSRI treatment actually had significantly better observed outcomes with non-SSRI treatment. Likewise, cases predicted by the HAP-SNP model to do well with SSRI treatment actually had significantly worse observed outcomes with non-SSRI drugs.

**References**

10. Lingjaerde O, Ahlfors UG, Bech P, Dencker SJ, Elgen K (1987) The UKU side effect rating scale. A new comprehensive rating scale for psychotropic drugs and a cross-sectional study of side effects in neuroleptic-treated patients. Acta Psychiatr Scand Suppl 334: 1-100.

11. Marchini J, Howie B, Myers S, McVean G, Donnelly P (2007) A new multipoint method for genome-wide association studies by imputation of genotypes. Nat Genet 39: 906-913.

**Table S9** SNPs most strongly associated with remission in SSRI treated derivation sample

| **Gene** | **Chromosome** | **Position*** | **SNP†** | ***P* value**‡ | **p value by controlling FDR** | **Genetic Mode** | **Ranking among 1400 SNPs** |
| --- | --- | --- | --- | --- | --- | --- | --- |
| *TPH2* | 12 | 70658496 | rs4760815 | 4.6×10^–4^ | 0.65 | Recessive | 1 |
| *SLC6A2* | 16 | 54252607 | rs3785143 | 4.8×10^–4^ | 0.34 | Allele | 2 |
| *GRM7* | 3 | 7661599 | rs1485161 | 7.1×10^–4^ | 0.33 | Dominant | 3 |
| *KCNN2* | 5 | 113805782 | rs10076582 | 8.7×10^–4^ | 0.30 | Dominant | 4 |
| *TPH2* | 12 | 70712221 | rs17110747 | 1.2×10^–3^ | 0.34 | Allele | 5 |
| *GRIA1* | 5 | 153007415 | rs729329 | 1.3×10^–3^ | 0.31 | Additive | 6 |
| *HTR7* | 10 | 92549888 | rs7916403 | 1.9×10^–3^ | 0.38 | Dominant | 7 |
| *GRM5* | 11 | 87938003 | rs1504096 | 2.2×10^–3^ | 0.38 | Genotype | 8 |
| *SLC6A2* | 16 | 54247926 | rs2242446 | 2.4×10^–3^ | 0.38 | Allele | 9 |
| *SLC6A2* | 16 | 54252052 | rs11076111 | 2.5×10^–3^ | 0.36 | Allele | 10 |
| *SLC6A4* | 17 | 25571040 | rs2020942 | 2.6×10^–3^ | 0.33 | Allele | 11 |
| *SLC6A4* | 17 | 25575791 | rs2066713 | 3.2×10^–3^ | 0.32 | Allele | 14 |
| *GRIK2* | 6 | 102157181 | rs572487 | 4.7×10^–3^ | 0.33 | Dominant | 20 |
| *TPH2* | 12 | 70663579 | rs11179027 | 5.3×10^–3^ | 0.33 | Recessive | 23 |
| *GRIK2* | 6 | 102158042 | rs543196 | 7.0×10^–3^ | 0.34 | Dominant | 29 |
| *TPH2* | 12 | 70650935 | rs17110532 | 0.01 | 0.36 | Recessive | 41 |
| *GAD1* | 2 | 171390986 | rs3828275 | 0.15 | 0.55 | Genotype | 368 |
| *GAD1* | 2 | 171379072 | rs12185692 | 0.33 | 0.66 | Genotype | 703 |

Abbreviations: SSRI, selective serotonin reuptake inhibitor; FDR, false discovery rate.

* Genomic position (NCBI Build 36).

† Underlined SNPs indicate top 10 SNPs strongly associated with SSRI response.

‡ Fisher’s exact test.

**Table S10** Association analysis results for depression diagnosis between normal controls and depressed patients of derivation sample for Top 10 SNPs significantly associated with SSRI response

| **Gene** | **Chromosome** | **Position*** | **SNP** | ***P* value†** | **p value by controlling FDR** | **Genetic mode** |
| --- | --- | --- | --- | --- | --- | --- |
| *TPH2* | 12 | 70658496 | rs4760815 | NA | NA | NA |
| *TPH2* | 12 | 70663579 | rs11179027 | NA | NA | NA |
| *GRIK2* | 6 | 102158042 | rs543196 | 0.75 | 0.78 | Recessive |
| *GAD1* | 2 | 171390986 | rs3828275 | 0.09 | 0.26 | Allele |
| *TPH2* | 12 | 70650935 | rs17110532 | 0.23 | 0.40 | Allele |
| *SLC6A4* | 17 | 25575791 | rs2066713 | 0.08 | 0.24 | Genotype |
| *GRIK2* | 6 | 102157181 | rs572487 | 0.90 | 0.91 | Additive |
| *TPH2* | 12 | 70712221 | rs17110747 | 0.13 | 0.30 | Recessive |
| *GAD1* | 2 | 171379072 | rs12185692 | NA | NA | NA |
| *SLC6A4* | 17 | 25571040 | rs2020942 | 0.18 | 0.36 | Genotype |

Abbreviations: SSRI, selective serotonin reuptake inhibitor; FDR, false discovery rate; NA, not applicable which indicates SNPs that could not be imputed due to the absence in the HapMap data.

* Genomic position (NCBI Build 36).

† Fisher’s exact test.

**Table S11** Genotypic combinations of polymorphism prediction model

|  | rs4760815 | rs543196 | rs3828275 | rs2066713 | *5-HTTLPR* | Predicted probability for response,  (number of patients) |
| --- | --- | --- | --- | --- | --- | --- |
| Predicted responder | AT+TT | CC | AA | CC | *ss* | >80%  (n=78) |
|  | AT+TT | CC | GG | CC | *ss* |  |
|  | AT+TT | CC | AA | CC | *sl*+*ll* |  |
|  | AT+TT | TC | AA | CC | *ss* |  |
|  | AT+TT | CC | GG | CC | *sl+ll* |  |
|  | AT+TT | TC | GG | CC | *ss* |  |
|  | AT+TT | CC | AG | CC | *ss* |  |
|  | AT+TT | CC | AA | TC+TT | *ss* |  |
|  | AA | CC | AA | CC | *ss* |  |
|  | AT+TT | CC | GG | TC+TT | *ss* |  |
|  | AT+TT | TC | AA | CC | *sl*+*ll* |  |
| Predicted nonresponder | AA | CC | AA | TC+TT | *sl*+*ll* |  |
|  | AT+TT | TT | AG | CC | *sl*+*ll* |  |
|  | AA | TC | AA | TC+TT | *ss* |  |
|  | AT+TT | TT | AA | TC+TT | *sl*+*ll* |  |
|  | AA | CC | GG | TC+TT | *sl*+*ll* |  |
|  | AA | TC | GG | TC+TT | *ss* |  |
|  | AA | TT | AA | CC | *sl*+*ll* |  |
|  | AT+TT | TT | GG | TC+TT | *sl*+*ll* |  |
|  | AA | TT | GG | CC | *sl*+*ll* |  |
|  | AA | CC | AG | TC+TT | *ss* |  |
|  | AT+TT | TC | AG | TC+TT | *sl*+*ll* |  |
|  | AT+TT | TT | AG | TC+TT | *ss* |  |
|  | AA | TC | AG | CC | *sl*+*ll* |  |
|  | AA | TT | AG | CC | *ss* | <30% |
|  | AA | TC | AA | TC+TT | *sl*+*ll* | (n=32) |
|  | AA | TT | AA | TC+TT | *ss* |  |
|  | AA | TC | GG | TC+TT | *sl*+*ll* |  |
|  | AA | TT | GG | TC+TT | *ss* |  |
|  | AA | CC | AG | TC+TT | *sl*+*ll* |  |
|  | AA | TC | AG | TC+TT | *ss* |  |
|  | AT+TT | TT | AG | TC+TT | *sl*+*ll* |  |
|  | AA | TT | AG | CC | *sl*+*ll* |  |
|  | AA | TT | AA | TC+TT | *sl*+*ll* |  |
|  | AA | TT | GG | TC+TT | *sl*+*ll* |  |
|  | AA | TC | AG | TC+TT | *sl*+*ll* |  |
|  | AA | TT | AG | TC+TT | *ss* |  |
|  | AA | TT | AG | TC+TT | *sl*+*ll* |  |
